# The evolving landscape of rheumatic drugs and their indications in paediatric and adolescent care

**DOI:** 10.1093/rheumatology/keaf455

**Published:** 2025-08-22

**Authors:** Coziana Ciurtin, Mihaela Sparchez, Despina Eleftheriou, Paul Brogan

**Affiliations:** Division of Medicine, Department of Ageing, Rheumatology and Regenerative Medicine, Centre for Adolescent Rheumatology, University College London, London, UK; Infection, Inflammation, Immunity Theme, NIHR University College London Hospitals Biomedical Research Centre, London, UK; 2nd Pediatric Discipline, Mother and Child Department, Iuliu Haţieganu University of Medicine and Pharmacy, Cluj-Napoca, Romania; Institute of Child Health, University College London, London, UK; Department of Paediatric Rheumatology, Great Ormond Street Hospital, London, UK; Institute of Child Health, University College London, London, UK; Department of Paediatric Rheumatology, Great Ormond Street Hospital, London, UK

**Keywords:** paediatric rheumatic drugs, targeted biologic treatments, small molecules, cell therapies, licensed therapies, toxicity

## Abstract

Paediatric rheumatic diseases (RMDs) are characterized by dysregulation of the immune system functions due to a combination of genetic, epigenetic and environmental factors. In many cases, children and young people (CYP) with RMDs require long-term pharmacological interventions to control their symptoms, minimize the risk of disease relapse and organ damage and ultimately preserve their quality of life. The past decades have seen significant progress in the expansion of therapeutic options licensed or available off-license for CYP with RMDs, and an unprecedented number of paediatric interventional clinical trials testing new therapies. This review aims to appraise the paediatric rheumatology community on available pharmacological therapies for use in childhood-onset RMDs, including conventional, biologic and targeted synthetic disease modifying anti-rheumatic drugs, immunoglobulins and cell-based therapies, highlighting their known indications, as well as current guidelines and consensus recommendations supporting their use off-license. We review the paediatric dosing regimens available for the treatment of RMDs and other autoimmune conditions, the toxicity profile of available therapeutic agents and provide a comprehensive evaluation of emerging therapies for childhood RMDs, currently tested in clinical trials.

Rheumatology key messages

- Recent research advances led to revised guidelines and recommendations for treatment of paediatric RMDs.
- A large number of biologic and targeted synthetic DMARDs are currently tested in clinical trials.
- Established and novel cell-based therapies hold the promise for long-term remission off-treatment in severe/refractory cases.
- Investment in equitable access to established/emerging therapies is needed to ensure better outcomes for children.

## Introduction

Notable progress has been achieved in recent decades in the treatment of rheumatic diseases (RMDs) due to significant industry and academic research investment in developing and testing new therapies. The main purpose of pharmacological intervention in paediatric rheumatology is to control the inflammatory processes underlying different clinical presentations, aiming for alleviation of symptoms, minimization of long-term damage or complications (including glucocorticoid toxicity), as well as preserving function and ensuring the maintenance of adequate quality of life, irrespective of the molecular mechanisms underpinning different pathologies.

Paediatric rheumatic conditions include monogenic autoinflammatory diseases (AID) characterized by key mutations affecting innate immune functions, multifactorial AID underpinned by interactions between genetic and environmental factors [1] and autoimmune rheumatic diseases (ARDs), typically involving the adaptive immune responses with a degree of overlap with innate immunity dysregulation [2].

RMDs that start early in life are broadly considered to have increased genetic burden. Recent research advances have facilitated better understanding of mechanisms of disease heritability and that of other complex molecular mechanisms contributing to disease pathogenesis, as well as facilitating novel biomarker discoveries enabling patient stratification within the same disease phenotype for improved management strategies [3].

We advocated that, based on genetic, molecular and clinical similarities, some RMDs should be classified in a similar way in children and adults, while conditions underpinned by significant differences require distinct childhood classification criteria [4] to enable timely recognition and diagnosis and support high-quality research and inclusion of homogeneous cohorts of children and adolescents in clinical trials.

Both the European Medicines Agency (EMA) and the Food and Drug Administration (FDA) recommend, where possible, testing of new adult therapeutic agents in children if they manifest with a similar clinical phenotype. These recommendations have facilitated many clinical trials in paediatric rheumatology leading to the licensing of new therapies for children with AID and ARDs. However, there is still a disconnect between paediatric and adult therapeutic indications, primarily related to the different disease labels across the lifespan, which sees many of the young people having their diagnosis changed when they transition into adulthood to fit adult-licensed treatment pathways, which limits the chance for high-quality research into long-term outcomes of RMDs with childhood onset.

In the UK, access to medications for children and young people (CYP) with rheumatic conditions is primarily mandated by the National Institute for Health and Care Excellence (NICE), the national health technology assessment body evaluating the cost-effectiveness of medicines granted marketing authorizations (‘product licences’) by the Medicines and Healthcare Products Regulatory Agency (MHRA) for medicines in the UK, and making them available on the National Health Service (NHS) through reimbursement. Additionally, access to drugs which are not NICE approved for use for paediatric rheumatology indications has been historically facilitated by the NHS England Medicines for Children Commissioning pathway, which enabled access to treatments for CYP aged <18 years old, where specific commissioning conditions within a NICE Technology Appraisal or NHS England clinical policy are met [5]. It is uncertain what process(es) will replace that when NHS England is fully decommissioned in October 2026, although it is likely to be similar, albeit sitting within the Department of Health and Social Care.

This review aims to update the rheumatology community on the current knowledge regarding the mechanism of action, doses and indications of the main therapies used in paediatric rheumatology, as well as review new emerging therapies currently tested in clinical trials for rheumatological indications and explore both established and emerging treatments that can potentially be used off-license in CYP with RMDs based on their licensing for other indications.

## Drugs used in paediatric rheumatology

Specialized therapeutic approaches for paediatric RMDs include the use of NSAIDs, intra-articular glucocorticoid (GC) injections (IAGC), systemic GC treatment and DMARDs, including conventional DMARDs (cDMARDs), biologic DMARDs [bDMARDs, comprising fusion proteins, monoclonal antibodies (mAb) or bi-specific mAb such as T-cell engagers], targeted synthetic DMARDs (tsDMARDs, also called ‘small molecules’), immunoglobulins and cell-based therapies. The last category includes hematopoietic stem cells (HSC) and mesenchymal stem cells (MSC, renamed medicinal signalling cells [6]), autologous or allogeneic haematopoietic stem cell transplant therapies [7] and more recently chimeric antigen receptor T-cell (CAR-T) therapies currently available only in clinical trials [8]. Although the terms immunosuppressant and immunomodulatory are used interchangeably, there are some important distinctions to consider. Immunosuppressants have a broader and non-selective impact on the immune functions and act by suppressing and dampening them to control inflammation associated with RMDs (e.g. most cDMARDs are immunosuppressant), while immunomodulatory therapies target more specific functions of the immune system, being designed to block certain molecules, cells or immune pathways and regulate and modulate their function, aiming to restore the immune homeostasis (bDMARDs, tsDMARDs or specific cell therapies are immunomodulatory).

## NSAIDs and GC

NSAIDs are frequently used as first-line therapy in JIA and management of acute musculoskeletal or glandular pain, which are frequent symptoms of childhood-onset RMDs [9]. They are recommended at the lowest effective dose for the shortest possible duration, although, in order to achieve their therapeutic benefit, NSAIDs need to be taken on a continuous scheduled basis for a duration of 4–8 weeks. Long-term use requires regular monitoring. The most commonly used NSAIDs are: ibuprofen (max. 30 mg/kg in three to four doses), naproxen (5–7.5 mg/kg twice daily, max. 500 mg, licensed from age 5), indometacin (0.5–1 mg/kg twice daily), diclofenac (1.5–2.5 mg/kg twice daily, max. 75 mg per dose), piroxicam (5–20 mg daily, as per body weight, licensed from age 6) and etoricoxib (60 mg once daily, increased if necessary to 90 mg once daily, licensed from age 16), while paracetamol is used from 30–60 mg every 8 h in very young children (maximum 60 mg/kg daily) to 0.5–1 g, every 4–6 h; maximum four doses per day in older adolescents.

The most common side effects of NSAIDs include gastrointestinal (GI) upset, rarely complicated with more severe manifestations such as GI bleeding. It is routine to use proton pump inhibitors alongside NSAIDs in children to minimize these side effects. Other side effects include haematuria, skin rashes, allergic reactions, accentuation of asthma symptoms and tinnitus. A recent study comparing ibuprofen with naproxen in children with JIA found similar efficacy, but increased side effects with naproxen [10], although this observation does not quite reflect the experience in practice where the less-frequent administration of naproxen coupled with its acceptable toxicity profile support its wide use.

Aspirin at a dose of 30–50 mg/kg/day is still recommended combined with intravenous immunoglobulin (IVIG) for Kawasaki disease (kDa) during the acute inflammatory phase, reducing to antiplatelet doses of 3–5 mg/kg/day when fever subsides [11]. Some studies suggested recently that the use of aspirin at 3–5 mg/kg/day from the outset is equally efficacious with potentially fewer side effects, but high-level evidence for this approach is lacking [12].

GC have been widely used for severe systemic manifestations of paediatric RMDs for decades, and despite attempts to minimize GC exposure they still are used widely for the treatment of children with RMDs. Pulse therapy with intravenous (IV) methyl-prednisolone at a dose of 30 mg/kg (max. 1 g/daily) for 3 days and/or oral prednisolone at a dose of 1–2 mg/kg once daily (max. 60 mg per dose) is recommended as the induction regimen for severe childhood-onset systemic lupus erythematosus (cSLE), including lupus nephritis (LN) [13]; severe inflammation or weakness associated with juvenile dermatomyositis (JDM) [14]; Takayasu arteritis (TA) [15]; polyarteritis nodosa (PAN); and for high-risk kDa presentation or in kDa cases with unsatisfactory response to IVIG [12]; childhood primary central nervous system vasculitis; or severe small vessel vasculitis, including granulomatosis with polyangiitis (GPA), microscopic polyangiitis (MPA) and eosinophilic granulomatosis with polyangiitis (EGPA) with significant organ involvement [15, 16]. A similar regimen has been historically used as first-line therapy in systemic-onset JIA (soJIA), recently renamed Still’s disease (comprising both soJIA and the adult-onset Still’s disease), although this should not delay early initiation of biologic therapy with interleukin (IL)-1 or IL-6 blockade as first-line therapy, which results in better outcomes [17]. High GC doses (IV pulsed methylprednisolone dose as above; or dexamethasone 10 mg/m2/daily IV) followed by oral prednisolone (1–2 mg/kg/day), in combination with IVIG or IL-1 inhibition, depending on aetiology and severity, are also recommended as first-line therapy in hemophagocytic lymphohistiocytosis (HLH)/macrophage activation syndrome (MAS) with persistent and severe inflammation and organ dysfunction [18, 19].

Following IV GC administration for induction regimens for paediatric RMDs with systemic manifestations and severe organ involvement, for the maintenance of disease control, GC dose should be tapered gradually in combination with other immunosuppressive therapies, to minimize the risk of GC toxicity. A paediatric glucocorticoid toxicity index (pGTI) has been developed and validated to assess the impact of morbidity related to GC exposure, which can be used in research and clinical practice [20, 21].

A consensus regimen for GC tapering has been proposed by the Childhood Arthritis and Rheumatology Research Alliance (CARRA) in collaboration with the Paediatric Rheumatology European Society (PReS) Lupus Working Party and the Paediatric Nephrology Research Consortium for the induction and maintenance therapy of proliferative LN, advocating for achieving a dose of 15 mg prednisolone daily or equivalent by week 12 [22]. Similarly, the European Alliance of Associations for Rheumatology (EULAR)/PReS recommendations for management of Still’s disease advocate for gradual tapering of oral GC at a dose of <0.2 mg/kg/day of prednisolone or equivalent and complete cessation of GC therapy after 6 months [17]. Short courses of oral GC are also recommended in the early inflammatory phase of juvenile localized scleroderma [23]. GC ‘bursts’ defined as maximum 2 mg/kg equivalent of prednisolone dose (maximum daily dose of 60 mg), with or without tapering over 6 weeks, were recommended by CARRA clinicians for chronic non-bacterial osteomyelitis (CNO), including recurrent multifocal osteomyelitis (CRMO) refractory to NSAIDs [24] and can also be used in periodic fever, aphthous stomatitis, pharyngitis and adenitis (PFAPA) syndrome or in IgA-related vasculitis [25].

Side effects of GC include mood swings, difficulty sleeping, weight gain, headaches, stomach pain and acne, hypertension, dyslipidaemia and increased glucose levels leading to increased cardiovascular risk, effects on growth and bone strength, delayed puberty, period abnormalities, impact on vaccine responses, increased risk of infections, while other side effects such as glaucoma, cataract or steroid-induced myopathy are less common in children. The efficacy as well as side effects of GC are treatment duration/dose-dependent and are explained by strong genomic effects (mediated by the activation of the cytosolic glucocorticoid receptor, cGCR) [26]. GC also have non-genomic effects through influencing the function of cell membrane proteins or mediated by proteins released from the cGCR–multiprotein complex following the binding of GC to the cGCR, which are considered responsible for some of the rapid effects of GC [26].

## Intra-articular administration of GC

IAGC injections are commonly used as first-line therapy in the management of JIA, aiming to bring joint inflammation under rapid control, provide symptomatic relief while allowing other therapies, such as MTX, to become effective. Out of all GC depot preparations available, triamcinolone hexacetonide (TH) is the option of choice, due to its unique characteristics, such as prolonged benefit, decreased systemic absorption and similar toxicity profile compared with more soluble GC preparations. When not available, triamcinolone acetonide (TA) can be used as an alternative, although its level of systemic absorption is higher than that of TH, in addition to having an increased rate of joint inflammation relapse [27]. The doses of TH that are usually used in paediatric population are 1 mg/kg per joint for large joints such as knees, shoulders or hips (max. 40 mg/joint); 0.5 mg/kg per joint for medium joints, such as ankles, subtalar joints, elbows and wrists (max. 30 mg/joint); and 0.04–1 mg in total dose per each small joint injected, such as fingers and toes [28].

IAGC injections for management of temporomandibular joint (TMJ) inflammation associated with JIA typically used a dose of 0.25 mg/kg per joint. Despite their recognized benefit [29], more recently there are concerns that they may have deleterious effects on TMJ growth during childhood [30] and current guidelines are that intra-articular glucocorticoid injection is not recommended as first-line management of TMJ arthritis in skeletally immature young people, but may be considered in cases refractory to optimized systemic treatment; or once skeletal maturity has been reached [31].

The side effects of IAGC injections depend on the dose. CYP who undergo multiple IAGC injections may experience short-lived side effects similar to those associated with systemic GC treatment. Additionally, IAGC injections are usually administered under local or general anaesthetic as age-appropriate, can in rare cases can cause joint infection, subcutaneous fat atrophy or incidental intra-articular calcification [28]. It is widely recommended that a specific joint should not be injected >3–4 times per year [32].

## Conventional DMARDs (cDMARDS)

Conventional disease-modifying anti-rheumatic drugs (cDMARDS) are widely used in RMDs in children despite the relatively poor-quality evidence for their efficacy in paediatric populations, as many disease-specific recommendations are based on data derived from clinical trials in adults with similar disease phenotypes. Many cDMARDs are used outside the licensed age where there are no good-quality studies in children (off-license), and also as unlicensed drugs, in refractory cases of paediatric RMDs, where no suitable licensed therapeutic alternatives are available for either adults or children (see Table 1).

**Table 1.** Conventional DMARDs used in paediatric RMDs

cDMARD/Mechanism of action/Type of molecule	Clinical indications the treatment is licensed for in children	Off-license use in children	Dosage recommendation	Side effects
Methotrexate Folic acid antagonist	pJIA, ≥2 years of age Metoject (subcutaneous formulation) is licensed from age 3.	Childhood psoriasis, Crohn’s disease, other forms of JIA, JDM, localized scleroderma, AAV, sarcoidosis, cSLE.	10–15 mg/m²/week, orally or subcutaneous or intramuscularly. In refractory cases, the weekly dosage may be increased to 20 mg/m²/week. Recommended to be prescribed with folic/folinic acid supplementation.	Nausea; vomiting; diarrhoea; stomach pain; mouth sores or ulcers; hair loss or thinning; headaches; dizziness; tiredness or fatigue; loss of appetite; increased sensitivity to sunlight. Serious side effects: liver damage (including cirrhosis); lung problems (such as inflammation or fibrosis); kidney damage (potentially leading to kidney failure); bone marrow suppression; severe skin reactions (like Stevens–Johnson syndrome); severe allergic reactions (anaphylaxis); increased risk of certain cancers, such as lymphoma. Contraindicated during conception, pregnancy and breastfeeding.
Leflunomide Pyrimidine synthesis inhibitor	Not licensed	Used off-license as alternative to methotrexate.	pJIA: loading dose to 100 mg daily for 3 days, followed by 10 mg/1.73 m^2^/day, which can be increased to 20 mg/1.73 m^2^/day, up to a max dose of 20 mg daily. It can be used either as monotherapy or in combination with methotrexate.	Diarrhoea; nausea; abdominal pain; hair loss; decrease in appetite/weight loss; rash; hypertension; asthenia; mouth ulcers; headache; hypersensitivity; abnormal liver function tests; lung involvement; bone marrow suppression; neuropathy. Contraindicated during conception, pregnancy and breastfeeding. Washout recommended in the context of severe side effects.
Sulfasalazine Anti-inflammatory and immunomodulatory effects, by inhibiting TNF-α expression, leucocyte accumulation and B-cell function	pJIA (≥6 years of age) UC (≥2 years of age)	Crohn’s disease.	JIA: Initial 10 mg/kg/day, increase weekly by 10 mg/kg/day, usual doses: 30–50 mg/kg/day in 2 divided doses, maximum dose: 2 g/day. UC: 30 mg/kg/day divided into 4 doses.	Headache; loss of appetite; rash; itching; fever; increased sensitivity to sunlight; orange-yellow discoloration of urine, sweat or tears (which can also stain soft contact lenses); dizziness; low sperm count in men (this is usually reversible upon stopping the medication); liver abnormalities; blood disorders. Rare: agranulocytosis.
Hydroxychloroquine Antimalarial Immunomodulatory and anti-inflammatory agent	Treatment and prophylaxis of malaria	JIA (in combination with other therapies), discoid lupus, cSLE, APS, cSjD.	≥31 kg: 5 mg/kg based on ideal body weight up to 400 mg, once a day.	Nausea; vomiting; diarrhoea; stomach pain; headache; dizziness; hair loss; rash; itching; skin discoloration (blue-black or gray); tinnitus; loss of appetite; ocular toxicity; vomiting; acute hepatic failure; agranulocytosis; QT interval prolongation.
Mycophenolate mofetil Antimetabolite immunosuppressant	Prophylaxis of organ rejection in paediatric recipients (≥3 months of age) and older of allogeneic kidney, heart or liver transplants.	AAV, severe and/or MTX-refractory juvenile localized scleroderma, cSLE, LN, systemic sclerosis, for primary systemic vasculitides.	SLE/PAN: 600 mg/m²/day (max. 1 g/day) for the first week, followed by 1200 mg/m²/day (maximum 2 g/day) in 2 divided doses. Kidney transplant: 600 mg/m2 orally twice daily, up to max. of 2 g daily. Liver transplant: 600 mg/m2 orally twice daily up to max. of 900 mg/m BD (3 g or 15 mL of oral suspension).	Diarrhoea; nausea; vomiting; stomach pain; headache; dizziness; fatigue; weakness; loss of appetite; rash; hair loss; high blood pressure; increased risk of severe infections; bone marrow suppression; liver damage; gastrointestinal bleeding or ulcers; an increased risk of certain cancers (such as lymphoma and skin cancer); progressive multifocal leukoencephalopathy (PML). Contraindicated during conception, pregnancy and breastfeeding.
Azathioprine Purine antimetabolite	Prevention and treatment of rejection in solid organ transplantation.	cSLE, JIA, JIA-associated uveitis, autoimmune hepatitis, inflammatory bowel disease, myasthenia gravis, LN, immune thrombocytopenia, AIHA, for primary systemic vasculitides.	1–2.5 mg/kg/day, single dose or a twice-daily schedule, maximum 200 mg/day.	Nausea; vomiting; diarrhoea; stomach pain; bone marrow suppression (leading to low white blood cell counts, anaemia and low platelets); increased risk of severe infections; hepatotoxicity; pancreatitis; flu-like symptoms; rash; hair loss; and an increased risk of skin cancer and lymphoma.
Tacrolimus Calcineurin-inhibitor	Systemic: prophylaxis of organ rejection patients receiving allogeneic liver, kidney, heart or lung transplant, with other immunosuppressants. Topical use: severe atopic dermatitis (0.03% ointment in ages ≥2 years; 0.1% ointment in ages ≥16 years).	Systemic: LN, refractory nephrotic syndrome, IgA vasculitis nephritis, Crohn’s disease, graft-*vs*-host disease, myasthenia gravis. Topical: active plaque morphoea, cutaneous cSLE.	0.15–0.3 mg/kg/day, divided in two doses, every 12 h. Target blood trough concentration: 5–20 ng/mL, 12 h after dose.	Nephrotoxicity; hepatotoxicity; neurotoxicity (tremor, headache, paraesthesia, seizures); hyperglycaemia; hypertension; hyperkalaemia; gastrointestinal symptoms (diarrhoea, nausea, vomiting, abdominal pain); alopecia; rash; insomnia; an increased risk of severe infections; and an increased risk of malignancy, particularly post-transplant lymphoproliferative disorder (PTLD).
Ciclosporin Calcineurin-inhibitor	Solid organ transplantation; bone marrow transplantation, endogenous uveitis, nephrotic syndrome, severe psoriasis, severe atopic dermatitis.	LN, JIA, JIA-associated uveitis, MAS, relapsed/refractory AIHA and refractory Kawasaki disease.	2–4 mg/kg/day in 2 divided doses, gradually increased to a maximum dose 6 mg/kg/day. Nephrotic syndrome: 6 mg/kg/day in 2 divided oral doses.	Nephrotoxicity; hypertension; hyperkalaemia; hyperuricemia; hepatotoxicity; gingival hyperplasia; hirsutism; neurotoxicity (e.g. headache, tremor, seizures, encephalopathy); increased susceptibility to infections; an increased risk of malignancy (particularly lymphoma and skin cancer); dyslipidaemia; gastrointestinal disturbances (e.g. nausea, vomiting, diarrhoea, abdominal pain); hypomagnesemia; and myalgia.
Etoposide Topoisomerase II inhibitor	Not licensed	HLH/MAS	50–150 mg/m2/dose 1–2 doses per week	Myelosuppression (leading to neutropenia, leukopenia, thrombocytopenia and anaemia); gastrointestinal toxicity (including nausea, vomiting, stomatitis, mucositis and anorexia); alopecia; hepatotoxicity; asthenia; hypersensitivity reactions (anaphylaxis); peripheral neuropathy; hypotension or hypertension; and an increased risk of secondary malignancies (acute myeloid leukaemia).
Cyclophosphamide Alkylating agents	Minimal change nephrotic syndrome in paediatric populations, malignant lymphomas.	Proliferative LN, neuropsychiatric lupus, primary systemic vasculitides, primary central nervous system vasculitis, JDM with severe extra-muscular or refractory disease or major organ involvement, juvenile SSc-related ILD, early diffuse cutaneous SSc with poor prognosis.	Nephrotic syndrome: 2 mg/kg/day oral dose for 8–12 weeks (maximum cumulative dose 168 mg per kg). LN (Low-dose: Euro-Lupus regimen): 300–500 mg/m^2^, IV maximum dose 500 mg, every 2 weeks for 6 doses. Organ- or life-threatening disease (high-dose: NIH protocol): 0.75- 1 g/m^2^/month IV (maximum 1 g) for 6 months. AAV (CYCLOPS regime): 15 mg/kg IV every 2 weeks for 3 pulses, then every 3 weeks for 7 pulses. JDM: 500–1000 mg/m^2^ monthly IV for 6 months. Juvenile SSc with ILD: 500 mg/m^2^ IV followed by monthly 750 mg/m^2^ for 6 months then every 2 months. PAN: 500 mg/m^2^ for first dose, IV then increase to 750 mg/m^2^/day (maximum dose 1.2 g) at weeks 0, 2, and 4 and then every 3 weeks until remission (maximum 10 IV doses and minimum 6 doses).	Nausea and vomiting; myelosuppression (leukopenia, anaemia, thrombocytopenia); alopecia; haemorrhagic cystitis; mucositis; stomatitis; amenorrhea; azoospermia; anorexia; asthenia; diarrhoea; abdominal pain; Cardiotoxicity (myocarditis, pericarditis, congestive heart failure); pulmonary toxicity (interstitial pneumonitis, pulmonary fibrosis); secondary malignancies (e.g. leukaemia, bladder cancer); infertility (permanent or temporary); veno-occlusive liver disease; anaphylaxis; severe skin reactions (e.g. Stevens–Johnson syndrome). Contraindicated during conception, pregnancy and breastfeeding.

AAV: ANCA-associated vasculitis; AIHA: autoimmune haemolytic anaemia; ANCA: anti-neutrophil cytoplasmic antibodies; APS: antiphospholipid syndrome; BSA: body surface area; cSLE: childhood-onset systemic lupus erythematosus; HLH: hemophagocytic lymphohistiocytosis; JDM: juvenile dermatomyositis; ILD: interstitial lung disease; LN: lupus nephritis; MAS: macrophage activation syndrome; MTX: methotrexate; NIH: National Institute of Health; PAN: polyarteritis nodosa; pJIA: polyarticular juvenile idiopathic arthritis; SSc: systemic sclerosis; UC: ulcerative colitis.

## Methotrexate

The best quality of evidence for dosing and efficacy of MTX in children is derived from studies in JIA. MTX is the most used cDMARD in paediatric rheumatology, being conditionally recommended over other cDMARDs for the treatment of oligoarticular JIA (oligoJIA) and TMJ arthritis [33] and for polyarticular JIA (pJIA) [34] by the American College of Rheumatology (ACR), with the subcutaneous formulation being preferred. The British Society of Rheumatology (BSR) guideline for management of psoriatic arthritis (PsA) across the life course also recommends MTX as the preferred cDMARD for management of juvenile PsA (JPsA) [35] despite low-quality evidence for efficacy [36]. The SHARE initiative also advocates for its use in JIA-associated uveitis associated with poor prognostic factors, as the first choice for systemic immunosuppression [37]. Only two randomized controlled trials (RCTs) evaluated the efficacy of MTX *vs* placebo in JIA overall [38] and specifically in extended-oligo articular and soJIA [39]. MTX is not effective and therefore not recommended in Still’s disease in children (soJIA) [33] or across the life course [17], although it is still used for polyarticular disease course, usually in combination with bDMARDs [40].

MTX is also used off-license in various other RMDs, including cSLE, JDM, anti-neutrophil cytoplasmic antibodies (ANCA)-associated vasculitides (AAV), localized scleroderma, sarcoidosis, as well as in childhood psoriasis or inflammatory bowel disease (Table 1). The main side effects CYP treated with MTX encounter are the GI upset and liver dysfunction. Overall, there is better MTX tolerability in younger children compared with adolescents [41]. To minimize the side effects to MTX, different strategies can be employed, such as optimization of the timing of the dose (e.g. administration at bedtime, evening, weekend) and route of administration (subcutaneous administration or liquid/syrup formulations); dietary adjustments (administration with food, adequate hydration); increase in the folic acid supplementation (up to 5 mg daily, 6 days a week, with the exception of the day of MTX administration) and use of antiemetic medications. Additionally, educational and behavioural approaches to tackle anticipatory nausea, combined with the change in route of MTX administration can help improve treatment tolerability [42].

## Other cDMARDs for use in JIA

There is scarce evidence for efficacy of other cDMARDs in paediatric populations, and many are used off-label across the world for various indications, especially in refractory disease with no other suitable therapeutic alternatives available (Table 1). The ACR guidelines for management of pJIA include leflunomide (LEF), sulfasalazine (SSZ) and hydroxychloroquine (HCQ) as potential alternative to MTX (in this order) [34], acknowledging the more recent evidence derived from RCTs which tested the efficacy of LEF [43, 44] and SSZ [45] in JIA, as well as the superiority of combination cDMARD therapy over MTX monotherapy for early JIA treatment [46]. Other DMARDs historically used in inflammatory arthritis such as penicillamine or gold therapy are not considered suitable treatment alternatives for JIA [47].

A recent study from the USA assessing commercially insured children identified a declining trend in the new use of cDMARDs in JIA, paralleled by an increasing trend in use of the bDMARDs [48], which reflects advances in therapeutic strategies in JIA.cDMARDs used in paediatric systemic RMDs

Various cDMARDs have been used in paediatric RMDs, including cSLE, JDM, systemic sclerosis, vasculitis or childhood-onset Sjögren’s disease (cSjD) associated with refractory manifestations or organ involvement, such as MMF, MTX, azathioprine (AZA), ciclosporin, tacrolimus (TAC) and cyclophosphamide (CYC). Based on the evidence derived primarily from adult studies and smaller, low-quality studies in paediatric populations, some cDMARDs are preferentially used for certain indications (see Table 1).

Historically, IV CYC therapy has been used with success in children with severe disease manifestations, and it is still recommended over oral CYC regimens due to reduced toxicity profile, for rapidly progressive RMDs, with significant organ involvement or poor prognostic factors, such as CNS lupus, LN [13], systemic vasculitides [15] or JDM [14]. MMF is superior to other cDMARDs as induction and maintenance regimen for class III and IV ± V LN [49, 50], and based on the merging evidence from recent clinical trials in adult LN, it is recommended for use in association with a calcineurin inhibitor (CNI), such as ciclosporin or TAC, which can be used off-license in children with poor prognostic factors and when the renal function is not severely impaired [49, 50].

AZA, MTX or MMF are recommended as first-line maintenance cDMARD therapy for paediatric systemic vasculitides [15]. The rates of remission induction in childhood PAN were similar with MMF and CYC in an open-label, randomized, Bayesian noninferiority trial, although MMF was associated with better health-related quality of life than CYC [51].

AZA, TAC, MTX and MMF can be used in JDM as first, second or third-line treatment options, with no preferential recommendation, because of lack of comparative evaluation of their efficacy. cDMARDs are not recommended for routine use in cSjD, unless there is evidence of specific-organ involvement [52]. MTX is the first-line cDMARD therapy for juvenile localized scleroderma, although other cDMARDs can also be used [23]. Etoposide and ciclosporin are also therapeutic alternatives for treatment of refractory HLH/MAS [18] (see Table 1).

MTX or SSZ are also used in CNO/CRMO refractory to NSAIDs or GC ‘bursts’ as per consensus recommendations [24]; MTX, SSZ, AZA, MMF or even CYC in childhood-onset sarcoid refractory to short courses of GC, mirroring adult practice [53]. cDMARDs should be prescribed and monitored according to existing age-appropriate national and international guidelines, taking into account safety aspects during conception, pregnancy and breast-feeding [54], which are potentially relevant to older adolescents who need to be counselled appropriately regarding pregnancy risk.

Table 1 includes details of licensed doses as well as the doses used off-license in children for various rheumatological indications, as well as their most commonly encountered side effects. Of note, only MTX and SSZ are licensed for use in JIA, while the majority of other cDMARDs are used off-license in paediatric RMDs.

## Biologic therapies in paediatric rheumatology

Undoubtedly, the greatest advance of the therapeutic armamentarium leading to improved outcomes in paediatric rheumatology has been achieved through the clinical implementation of biologic immunosuppressive therapies, which only became widely available in the last decades, with the first biologic receiving FDA approval for use in JIA in 1999.

Biologic agents are genetically engineered proteins that selectively block individual components of the immune system and inflammatory pathways responsible for chronic inflammation, developed using recombinant DNA technology. The research underpinning their discovery has also revolutionized the understanding of the pathogenesis of many paediatric RMDs.

Overall, bDMARDs are currently recommended for the treatment of JIA, including Still’s disease (soJIA), JIA-associated uveitis, AIDs and connective tissue diseases such as cSLE and JDM, with some of them having the advantage of being better characterized in terms of both safety and efficacy than many cDMARDs used in CYP with RMDs, following successful completion of RCTs in JIA [55], cSLE [56] or AAV [57].

However, there are still unknown aspects related to their life-long efficacy and safety, hampered by the lack of long-term follow-up of cohorts from paediatric to adult rheumatology services. Additionally, despite the availability of many bDMARDs, CYP with JIA experienced high rates of relapse following treatment discontinuation after achieving remission, both in clinical trials [58] and in real life [59], suggesting that future research is still required to identify biomarkers with adequate flare predictive value in clinical practice [60] and support the development of effective therapeutic strategies to ensure the profound remission in JIA [61] for ultimate patient benefit.

There is also little consensus on the best approaches to tackle immunogenicity leading to secondary bDMARD treatment failure in CYP who develop anti-drug neutralizing antibodies [62], as pro-active drug monitoring strategies are not widely implemented in paediatric rheumatology clinical practice, as the impact is questionable [63].

Despite these limitations, bDMARDs have changed the paradigm of treatment of many paediatric RMDs, being currently recommended as a first-line DMARD therapy in severe presentations associated with Still’s disease [17], AAV [15, 64], Takayasu arteritis [15], HLH-MAS [18] or in combination with cDMARDs as first-line therapy for LN associated with poor prognostic factors [50] across the lifespan.

## Biologics for JIA

Anti-tumour necrosis factor (TNF)-α inhibitors (TNFi) are the most used therapeutic agents for non-systemic JIA refractory to cDMARD therapy, due to their proven efficacy across various JIA subtypes [65]. The TNFi currently licensed in JIA are adalimumab [66] and golimumab [67] (both human monoclonal antibodies) and etanercept [68], a soluble TNF receptor fusion protein. Infliximab (a chimeric monoclonal antibody) has also been successfully used off-label in JIA [69]. The mechanism of action of TNFi, licensed indications or off-label recommendations and corresponding dosage regimens are detailed in Table 2.

**Table 2.** Biologic DMARDs and biosimilars licensed for use in children

bDMARDs	Mechanism of action	Clinical indications the treatment is licensed for in children	Off-license use in children	Dosage recommendation
TNF-α blockade				
Adalimumab	Human monoclonal antibody against TNF-α	pJIA (≥2 years) ERA (≥6 years) Uveitis (≥2 years) Crohn’s disease (≥6 years)	Severe/refractory JDM, sarcoid, CRMO	JIA: 10 mg EOW (10 to <15 kg) 20 mg EOW (15 to <30 kg) 40 mg EOW (≥30 kg) Crohn’s disease: Initial doses: 80 mg on day 1, then 40 mg 2 weeks later (17 to <40 kg) 160 mg on day 1, then 80 mg 2 weeks later (≥40 kg) Maintenance dose: 20 mg EOW (17 to <40 kg); 40 mg EOW (≥40 kg)
Etanercept	Soluble TNFα receptor fusion protein	pJIA (≥2 years) JPsA (≥2 years) ERA (≥12 years) Plaque psoriasis (≥4 years)	N/A	0.4 mg/kg twice weekly 0.8 mg/kg EW 50 mg EW
Infliximab	Chimeric monoclonal antibody against TNF-α	Crohn’s disease (≥6 years) Ulcerative colitis (≥6 years)	pJIA, sarcoid, uveitis, JDM	5–10 mg/kg IV as an induction regimen at 0, 2 and 6 weeks, followed by a maintenance regimen of 5–10 mg/kg Q8W
Golimumab	Human monoclonal antibody against TNF-α	pJIA (≥2 years) JPsA (≥2 years)	N/A	50 mg Q4W
Co-stimulatory blockade				
Abatacept	Human CTLA4 fusion protein co-stimulatory blockade	Licensed for pJIA (≥2 years)	Severe/refractory JDM	<75 kg: 10 mg/kg IV at 0–2–4 weeks and Q4W thereafter >75 kg, adult doses are recommended: 60 kg–100 kg–750 mg >100 kg–1000 mg
IL-1 blockade				
Anakinra	Human recombinant IL-1 receptor antagonist IL-1β and IL-1α blockade	CAPS, FMF, Still’s disease From 8 months of age and weighing at least 10 kg	N/A	<50 kg: 1–2 mg/kg/day SC >50 kg: similar to adult doses, 100 mg daily Severe CAPS: 3–4 mg/kg/day, which can be adjusted to a maximum of 8 mg/kg/day
Canakinumab	Human monoclonal antibody Anti IL-1β	Still’s disease (≥2 years) CAPS (≥4 years) TRAPS: adult and paediatric patients MKD: adult and paediatric patients FMF: adult and paediatric patients	N/A	Still’s disease (SoJIA): ≥7.5 kg: 4 mg/kg (maximum dose of 300 mg) SC Q4W CAPS: >40 kg: 150 mg subcutaneously, every 8 weeks ≥15 kg and ≤40 kg: 2 mg/kg SC Q8W 15 kg to 40 kg with an inadequate response, the dosage can be increased to 3 mg/kg SC Q8W TRAPS, HIDS/MKD and FMF: >40 kg: 150–300 mg subcutaneously, Q4W ≤40 kg: 2–4 mg/kg SC Q4W
Rilonacept	Dimeric fusion protein of human IL-1 receptor (IL-1R1) and IL-1 receptor accessory protein (IL-1RAcP) IL-1β and IL-1α blockade	Licensed for CAPS (≥12 years)	N/A	Loading dose of 4.4 mg/kg, up to a max. 320 mg SC Then 2.2 mg/kg, up to a max. 160 mg SC EW
IL-6 blockade				
Tocilizumab	Human monoclonal antibody Anti IL-6 receptor	Licensed for: Still’s disease (≥2 years pJIA (≥2 years)	Severe juvenile systemic sclerosis, JDM.	pJIA: <30 kg: 10 mg/kg IV Q4W 162 mg SC Q3W ≥30 kg: 8 mg/kg IV Q4W 162 mg SC Q2W Still’s: <30 kg: 12 mg/kg IV Q2W 162 mg SC Q2W ≥30 kg: 8 mg/kg IV Q2W 162 mg SC EW
IL-17 blockade				
Secukinumab	Human monoclonal antibody Anti IL-17A	ERA (≥4 years) JPsA (≥2 years) Plaque psoriasis (≥6 years)	N/A	ERA, JPsA, plaque psoriasis: ≥15 kg and <50 kg: 75 mg SC ≥50 kg: 150 mg SC Weeks 0,1, 2, 3 and 4 and then Q4W
Ixekizumab	Human monoclonal antibody Anti IL-17A	Plaque psoriasis (≥6 years)	JPsA	>50 kg: 160 mg SC (two 80 mg injections) at week 0, then 80 mg Q4W 25–50 kg: 80 mg SC at week 0, then 40 mg Q4W <25 kg: 40 mg SC at week 0, then 20 mg Q4W
IL12/23 blockade				
Ustekinumab	Human monoclonal antibody Anti IL-12 and IL-23 (p40)	JPsA (≥6 years) Plaque psoriasis (≥6 years)	N/A	<60 kg: 0.75 mg/kg SC at Weeks 0, 4 and then Q12W 60–100 kg: 45 mg SC at Weeks 0, 4 and then Q12W >100 kg: 90 mg SC at Weeks 0, 4 and then Q12W
IFN blockade				
Emapalumab	Human monoclonal antibody Anti IFNγ	Licensed for: Newborn and older children with primary HLH with refractory, recurrent or progressive disease or intolerance with conventional HLH therapy (FDA approval) MAS in Still’s disease with an inadequate response or intolerance to glucocorticoids, or with recurrent MAS (FDA approval)	N/A	HLH: Starting dosage: 1 mg/kg as an IV infusion over 1 h twice per week (every three to four days) Still’s disease and MAS: 6 mg/kg load; then 3 mg/kg every 72 h Can increase to 10 mg/kg every 72 h if required
CD20 blockade				
Rituximab	Chimeric monoclonal antibody Anti CD20	GPA (≥2 years) MPA (≥2 years) Mature B cell NHL (≥6 months) Mature B-cell acute leukaemia (≥6 months)	pJIA, refractory/severe cSLE, JDM, systemic sclerosis, cSjD, monogenic lupus, refractory HLH/MAS	GPA and MPA: The induction dose, in combination with glucocorticoids: 375 mg/m^2^ IV weekly for 4 weeks The follow-up dose, in combination with glucocorticoids: two 250 mg/m^2^ IV infusions separated by two weeks, followed by a 250 mg/m^2^ IV infusion every 6 months thereafter based on clinical evaluation pJIA: 375 mg/m^2^ IV infusion two doses, 2 week apart per course
BAFF blockade				
Belimumab	Human monoclonal antibody anti-BAFF	cSLE (≥ 5 years) Lupus nephritis (≥ 5 years) Limitations of Use: The efficacy of Belimumab has not been evaluated in patients with severe active CNS lupus	cSjD with glandular manifestations, monogenic lupus	IV: 10 mg/kg at 2-week intervals for the first 3 doses and then Q4W SC: ≥40 kg, 200 mg EW SC: 15–40 kg, 200 mg E2W
Biosimilars				
Etanercept-SZZS Etanercept-YKRO Benepali Nepexto	Soluble TNFα receptor fusion protein	JIA (pJIA, extended oligoarthritis, JPsA, ERA) – (≥2 years) Plaque psoriasis(≥6 years)	N/A	0.8 mg/kg EW, maximum dose 50 mg per week
Adalimumab-ADAZ Adalimumab-ATTO Adalimumab-AACF Adalimumab-FKJP Adalimumab-AATY Adalimumab-AFZB	Human monoclonal antibody against TNF-α	pJIA (≥2 years) ERA (≥6 years) Uveitis (≥2 years) Crohn’s disease (≥6 years)	Severe/refractory JDM, sarcoid, CRMO	JIA: 10 mg EOW (10 to <15 kg) 20 mg EOW (15 to <30 kg) 40 mg EOW (≥30 kg) Crohn’s disease: Initial doses: 80 mg day 1, then 40 mg 2 weeks later (17 to <40 kg) 160 mg on day 1, then 80 mg 2 weeks later (≥40 kg) Maintenance dose: 20 mg EOW (17 to <40 kg); 40 mg EOW (≥40 kg)
Infliximab-AXXO Infliximab-DYYB Infliximab-QBTX Infliximab-ABDA	Chimeric monoclonal antibody against TNF-α	Crohn’s disease (≥6 years) Ulcerative colitis (≥6 years)	pJIA, sarcoid, uveitis, JDM	5 mg/kg IV as an induction regimen at 0, 2 and 6 weeks, followed by a maintenance regimen of 5 mg/kg Q8W
Rituximab-ABBS Rituximab-PVVR Rituximab-ARRX	Chimeric monoclonal antibody Anti CD20	GPA (≥2 years) MPA (≥2 years) Mature B-cell NHL (≥6 months) Mature B-cell acute leukaemia (≥6 months)	pJIA, refractory/severe cSLE, JDM, systemic sclerosis, cSjD, monogenic lupus, refractory HLH/MAS	GPA and MPA: The induction dose, in combination with glucocorticoids: 375 mg/m^2^ IV weekly for 4 weeks The follow-up dose, in combination with glucocorticoids: two 250 mg/m^2^ IV infusions separated by two weeks, followed by a 250 mg/m^2^ IV infusion every 6 months thereafter based on clinical evaluation. pJIA 375 mg/m^2^ IV infusion two doses, 2 week apart per course
Tocilizumab-ANOH Tocilizumab-BAVI Tocilizumab-AAZG	Human monoclonal antibody against IL-6 receptor	Licensed for: Still’s disease (≥2 years) pJIA (≥2 years)	Severe/refractory JDM	pJIA: <30 kg: 10 mg/kg IV Q4W 162 mg SC Q3W ≥30 kg: 8 mg/kg IV Q4W 162 mg SC Q2W Still’s disease: <30 kg: 12 mg/kg IV Q2W 162 mg SC Q2W ≥30 kg: 8 mg/kg IV Q2W 162 mg SC EW
Other therapies				
Intravenous immunoglobulins (IVIG), 10%		Replacement therapy (0–18 years) in: Primary humoral immunodeficiencies (PID) Secondary immunodeficiency syndromes (SID) – severe/recurrent infections, ineffective antimicrobial treatment and either proven specific antibody failure or serum IgG level of <4 g/l Immunomodulation (0–18 years) in: Primary immune thrombocytopenia (ITP) Guillain-Barré syndrome Kawasaki disease Chronic inflammatory demyelinating polyneuropathy (CIDP) Multifocal motor neuropathy (MMN)	Rapidly progressive/refractory JDM.	PID: starting dose: 0.4–0.8 g/kg; maintenance dose: 0.2–0.8 g/kg every 3–4 weeks SID: 0.2–0.4 g/kg, every 3–4 weeks. ITP: two alternative treatment schedules: 0.8–1 g/kg given on day 1; this dose may be repeated once within 3 days 0.4 g/kg given daily for 2–5 days. Guillain-Barré syndrome: 0.4 g/kg bw/day over 5 days Kawasaki disease: 2 g/kg as a single dose; in association with acetylsalicylic acid. CIDP: 2 g/kg divided over 2–5 consecutive days followed by maintenance doses of 1 g/kg over 1–2 consecutive days every 3 weeks. MMN: starting dose: 2 g/kg given over 2–5 consecutive days; maintenance dose: 1 g/kg every 2–4 weeks or 2 g/kg every 4–8 weeks.
Iloprost	Synthetic prostacyclin analogue	Not licensed in children	Idiopathic or familial pulmonary arterial hypertension (PAH) and Raynaud’s	PAH (8–17 years): initially 2.5 micrograms for 1 dose, increased to 5 micrograms for 1 dose, increased if tolerated to 5 micrograms 6–9 times a day, adjusted according to response; reduced if not tolerated to 2.5 micrograms 6–9 times a day, reduce to lower maintenance dose if high dose not tolerated. Raynaud’s (12–17 years): initially 0.5 nanogram/kg/min, increased to 1–2 nanograms/kg/min given over 6 h daily for 3–5 days, dose increase should be performed gradually.

CAPS: cryopyrin-associated periodic syndromes; CNS: central nervous system; CTLA4: cytotoxic T-lymphocyte-associated antigen 4; EMA: European Medicines Agency; EOW: every other week; EW: every week; FDA: Food and Drug Administration, USA; FMF: familial Mediterranean fever; GPA: granulomatosis with polyangiitis; HLH: hemophagocytic lymphohistiocytosis; IFN: interferon; IV: intravenously; JIA: juvenile idiopathic arthritis; JPsA: juvenile psoriatic arthritis; MAS: macrophage activation syndrome; MKD: hyperimmunoglobulin D syndrome (HIDS)/mevalonate kinase deficiency; MPA: microscopic polyangiitis; SC: subcutaneously; TRAPS: tumour necrosis factor receptor (TNF) associated periodic syndrome; pJIA: polyarticular juvenile idiopathic arthritis; Q4W: every 4 weeks; Q8W: every 8 weeks; Q12W: every 12 weeks.

There are several agents blocking IL-1 licensed for use in children (anakinra, canakinumab and rilonacept). Anakinra and canakinumab have revolutionized the outcomes of Still’s disease in children (soJIA), being currently recommended as first-line DMARD therapy (similarly to IL-6 blockade with tocilizumab) as early as possible after the diagnosis is made, even in CYP with risk factors for developing Still’s lung disease as per data currently available [17]. IL-1 blockade is also indicated for treatment of AIDs, such as cryopyrin-associated periodic syndromes (CAPS), familial Mediterranean fever (FMF) [70], TNF receptor-associated periodic syndrome (TRAPS) and mevalonate kinase deficiency [70–72] and in HLH/MAS [18, 19]. Rilonacept is also licensed for CAPS but rarely used in Europe.

Pro-inflammatory cytokine therapeutic blockade targeting IL-6 (tocilizumab) [73], IL-17A (secukinumab) [74] and IL12/23 (ustekinumab) [75], in addition to T-cell costimulatory blockade (abatacept) [76], have subsequently received license for treatment of specific JIA phenotypes (see Table 2). Ixekizumab (IL17A blockade) has shown benefit in JPsA as per preliminary data from the COSPIRIT-JIA trial (not published as yet).

B-cell targeted therapies such as CD20 blockade with rituximab has also been used off license in seropositive pJIA [77].

Multiple biosimilars, defined as biologic molecules which are similar in quality, safety and efficacy with their originator, have been developed and tested over time in JIA, and they have been proven equivalent with their originators in real-life clinical practice [78], successfully contributing to improved treatment accessibility (see Table 2).

## Biologics for paediatric RMDs

B-cell depletion with rituximab (anti CD20) is currently recommended as first-line DMARD therapy in severe/refractory GPA and MPA with childhood onset [15, 64] based on the data derived from a successful phase IIa clinical trial [57], which is one of the major advances in management of paediatric RMDs. Rituximab administered every 4–6 months is also superior to azathioprine for maintenance of remission in AAV, and is thus recommended [16, 79].

Belimumab (anti BAFF) is the first biologic to be licensed for use in cSLE after meeting the primary end point in the PLUTO trial [56], and is currently recommended to be used in combination with cDMARDs as first-line therapy for class III/IV LN with poor prognosis [49] and for refractory cSLE with various manifestation as thrombocytopaenia, cutaneous, musculoskeletal and systemic vasculitis as per recommendations available for adults [80], as the SHARE recommendation for management of cSLE require updating [13].

Biologic therapies have also been used off-license and are currently recommended by paediatric guidelines for use in other RMDs with severe and refractory presentations, based on data available form adult studies and lower quality evidence from paediatric cases (see Table 2). Abatacept and rituximab are recommended for use in severe/refractory JDM [14]; tocilizumab and TNFi as second or third-line therapeutic agents for induction and/or maintenance of severe paediatric vasculitides [15]; belimumab and rituximab for glandular manifestations in cSjD, and tocilizumab and rituximab for severe skin fibrosis or interstitial lung disease (ILD) in juvenile systemic sclerosis, both based on adult recommendations [52, 81]; rituximab for refractory HLH/MAS [18]; TNFi for refractory CNO/CRMO [24] and juvenile systemic granulomatosis with genetic causes, including early-onset sarcoidosis and Blau syndrome, as well as refractory sarcoidosis, irrespective of age an onset [53].

IVIG treatment is recommended as first-line therapy for kDa [11], for refractory JDM [14] and as first-line therapy of HLH/MAS associated with infection, in combination with GC and anakinra [18]. Emapalumab, a human mAb against interferon (IFN)γ is indicated (but not yet widely available in the UK or Europe) in primary HLH and MAS, supported by successful clinical trial data in children [82] or in HLH associated with Ebstein-Barr virus infection [18], and anifrolumab, a type I IFN receptor antagonist although not licensed in children, is emerging as potentially efficacious (off-license) in the management of autoinflammatory type I interferonopathies [83, 84].

Iloprost is a synthetic prostacyclin analogue, used off-license in CYP with digital ulcers secondary to vasculopathy and pulmonary hypertension based on indications available in adults [81]. Therapeutic plasma exchange is not routinely recommended but can be used off-license on a case-by-case basis in AAV [16, 79].

## Side effects of biologic DMARDs

The most common side effects of targeted biologic therapies are related to the blockade of the immune system to reduce inflammation, which can increase the susceptibility to infective complications, including viral, fungal and bacterial infections [85]. Screening to exclude chronic viral hepatitis, human immunodeficiency virus (HIV) infection or tuberculosis, vaccination in younger children before starting treatment or evidence for immunity to varicella and measles in older children, are all recommended before starting therapy, while life vaccines are contraindicated while on biologics [86]. Other common side effects include injection-site reactions for drugs with subcutaneous administration and infusion reactions for the one administered intravenously, in addition to laboratory abnormalities, including elevated liver enzymes and low blood-cell counts (with neutropenia being the most frequent), various GI manifestations and headache [87]. With the expansion of the biologic treatment options and their broader use, it became apparent that the selective blocking of the immune system could recapitulate some of the phenotypes of inborn errors of autoimmunity clinicians need to be aware of [88, 89].

## Synthetic targeted DMARDs (small molecules) used in paediatric rheumatology

Based on the recent successful clinical trials, tofacitinib, baricitinib and upadacitinib have received approval to be used as monotherapy or in combination with MTX for the treatment of active polyarticular course JIA, including rheumatoid factor (RF) positive and RF negative JIA, extended oligo JIA and juvenile PsA in CYP in which one or more cDMARD or bDMARD has/have been proven inadequate or not well tolerated [90–92]. Baricitinib is also licensed for use in refractory JIA-associated uveitis [93] and enthesitis-related arthritis (ERA) [91] refractory to DMARDs therapy.

JAKi are also recommended for treatment of interferonopathies as per EULAR/ACR ‘points to consider’ guideline [83] and evidence of clinical efficacy [94, 95]. JAKi can offer a suitable therapeutic alternative for refractory JDM [96], particularly if associated with calcinosis [97], with a RCT comparing the efficacy of baricitinib *vs* MTX in JDM (the BARJDM trial) currently in set-up [98].

Apremilast, a phosphodiesterase type-4 inhibitor licensed for use in adult PsA, is also recommended for use off-license in juvenile PsA by the BSR guideline [35], while trials in children are ongoing in Behçet’s disease (see Table 3).

**Table 3.** Targeted synthetic DMARDs and other small molecules

Synthetic targeted small molecules Mechanism of action/type of molecule	Clinical indications the treatment is licensed for in children	Off-license use in children	Dosage recommendation	Side effects
Tofacitinib JAK 1/3 inhibition	pJIA or JPsA (≥2 years of age)	Refractory JDM Moderate/severe alopecia areata Refractory uveitis/scleritis	10 kg ≤ body weight < 20 kg: 3.2 mg (3.2 mL oral solution) twice daily 20 kg ≤ body weight < 40 kg: 4 mg (4 mL oral solution) twice daily Body weight ≥ 40 kg: 5 mg (one 5 mg tablet or 5 mL oral solution) twice daily	Described more in JIA: abdominal pain; anaemia; cough; fever; gastrointestinal disorders; headache influenza; joint disorders; nausea; pharyngitis; vomiting. Described in all ages: increased risk of infection; diarrhoea; dyspepsia; fatigue; sinusitis; skin reactions; hypertension. Contraindicated during conception, pregnancy and breastfeeding.
Baricitinib JAK 1/2 inhibition	pJIA (RF+ or RF−) Extended oligoJIA ERA or JPsA Moderate/severe alopecia areata (≥2 years of age)	Refractory/severe JDM Autoinflammatory interferonopathies	JIA 10–30 kg: 2 mg/day once daily ≥30 kg: 4 mg/day once daily Monogenic interferonopathies <20 kg: 2 mg 4 times a day 20–40 kg: 4 mg twice daily >40 kg: 6 mg am, 4 mg pm (10 mg/day)	Abdominal pain; dyslipidaemia; headache; herpes zoster (interrupt treatment); increased risk of infection; nausea; skin reactions; thrombocytosis. Dose reduction is required if GFR <120 ml/min/1.73 m^2^. Contraindicated during conception, pregnancy and breastfeeding.
Upadacitinib Selective JAK 1 inhibition	Active pJIA, JPsA (≥2 years of age) Refractory, moderate/severe atopic dermatitis, (≥12 years of age)	N/A	pJIA/JPsA 10 kg to <20 kg – 3 mg (3 mL oral solution) twice daily 20 kg to <30 kg – 4 mg (4 mL oral solution) twice daily 30 kg and greater – 6 mg (6 mL oral solution) twice daily Atopic dermatitis Body weight ≥40 kg – 15 mg once daily If an adequate response is not achieved, the dose may be increased to 30 mg once daily	Abdominal pain; anaemia; cough; dyslipidaemia; fatigue; fever; headache; increased risk of infection; lymphopenia; nausea; neoplasms; neutropenia; skin reactions; weight gain. Contraindicated during conception, pregnancy and breastfeeding.
Apremilast Phosphodiesterase type-4 inhibitor	Paediatric psoriasis, (≥6 years of age and above 20 kg)	JPsA Ulcers associated with BD	20 mg twice daily for those weighing 20 kg to <50 kg; 30 mg twice daily for those weighing 50 kg or more	Headache, diarrhoea, stomach pain; vomiting; weight loss; signs of a common cold; back pain; joint pain; allergic reactions; increased risk of infection; insomnia. Contraindicated during conception, pregnancy and breastfeeding.
Dapaglifozin SGLT-2 inhibitor	Type II diabetes	LN	10 mg daily From age 10 years	Contraindicated in ketoacidosis; back pain; cystitis; dizziness; dyslipidaemia; hypoglycaemia (in combination with insulin or sulfonylurea); increased risk of infection; prostatitis; skin reactions; urinary and vulvovaginal disorders. The treatment should be discontinued when pregnancy is detected, not recommended for the 2nd and 3rd trimester of pregnancy.
Other treatments used off-license in paediatric rheumatology				
Pilocarpine Muscarinic receptor agonist (M1, M2, M3)	Not licensed in children	cSjD with symptoms of dryness	5 mg daily, increased to twice/three times daily based on effect and tolerability	Diarrhoea; headache; hyperhidrosis; hypersalivation; nausea; skin reactions; vision disorders; vomiting. Risk in pregnancy not known.
Sildenafil Phosphodiesterase type 5 (PDE-5) inhibitor	Pulmonary hypertension from 1 month of age	Severe Raynaud’s Digital ulcers	Child 1–17 years (body-weight up to 20 kg) 10 mg 3 times a day Child 1–17 years (body-weight 20 kg and above) 20 mg 3 times a day	Alopecia; anaemia; anxiety; cough; diarrhoea; dizziness; fluid retention; gastrointestinal discomfort; gastrointestinal disorders; headaches; increased risk of infection; insomnia; nasal complaints; nausea; night sweats; pain; skin reactions; tremor; vasodilation; vision disorders.
Bosentan Dual endothelin receptor antagonist	Pulmonary hypertension from 1 month of age	Severe Raynaud’s Digital ulcers	Child 2–17 years (body-weight 10–20 kg) Initially 31.25 mg once daily for 4 weeks, then increased to 31.25 mg twice daily Child 2–17 years (body weight 20–40 kg) Initially 31.25 mg twice daily for 4 weeks, then increased to 62.5 mg twice daily Child 12–17 years (body-weight 40 kg and above) Initially 62.5 mg twice daily for 4 weeks, then increased to 125 mg twice daily (max. per dose 250 mg)	Anaemia; abnormal liver function tests; diarrhoea; erythema; fluid retention; flushing; gastro-oesophageal reflux disease; headache; hypotension; nasal congestion; oedema; palpitations; syncope; leukopenia; neutropenia; thrombocytopenia; angioedema; abdominal pain; fever; increased risk of infection; pulmonary oedema; vision blurred; vomiting. Contraindicated during conception, pregnancy and breastfeeding.
Pamidronate Zolendronate Bisphosphonates, synthetic analogues of pyrophosphate, which are adsorbed onto hydroxyapatite with role in reducing the rate of bone turnover	Not licensed for use in children	CRMO Low bone density and fractures, as well as osteogenesis imperfecta	Pamidronate: 15–60 mg IV, as a single infusion or in divided doses over 2–4 days, dose adjusted according to body weight; maximum 90 mg per course. Most experience in CRMO: 1 mg/kg, max 60 mg per dose monthly or 1 mg/kg/dose, 3 consecutive days, every 3 months Zolendronate: initial 0.0125–0.025 mg/kg/dose every 6 months, can be increased to 0.05 mg/kg/dose (max 4 mg) (dependent on age) for CRMO, osteogenesis imperfecta and juvenile osteoporosis	Hypocalcaemia; flu-like symptoms; alopecia; anaemia; appetite decreased; arthralgia; constipation; diarrhoea; dizziness; dysphagia; electrolyte imbalance; eye inflammation; fever; gastritis; gastrointestinal discomfort; headache; influenza like illness; malaise; myalgia; nausea; oesophageal ulcer (discontinue); oesophagitis (discontinue); pain; peripheral oedema; renal impairment; skin reactions; taste altered; vomiting. Where exposure has occurred, either prior to or during pregnancy, skeletal development and neonatal calcium levels may be warranted.
Avacopan Complement 5a receptor (C5aR) antagonist	Not licensed for use in children	To be considered in active AAV (off-license) to reduce GC- toxicity	Dose recommended in adults with AAV: 30 mg BD Paediatric trial ongoing	Abdominal pain upper; abnormal liver function tests; diarrhoea; headache; increased risk of infection; leukopenia; nausea; neutropenia; vomiting; angioedema. Contraindicated during conception, pregnancy and breastfeeding.

BD: Behçet’s disease; CRMO: chronic recurrent multifocal osteomyelitis; cSjD: childhood-onset Sjogren’s disease; ERA: enthesitis related arthritis; JDM: juvenile dermatomyositis; JPsA: juvenile psoriatic arthritis; pJIA: polyarticular juvenile idiopathic arthritis; PsA: psoriatic arthritis; SGLT-2: sodium-glucose cotransporter-2.

## Tapering of DMARDs

A few paediatric studies explored biologic DMARD tapering strategies in JIA and provided evidence that the duration of JIA remission of minimum 1.5–2 years was usually associated with better outcomes [99], although overall, a high proportion of JIA cases flared post treatment withdrawal [59], highlighting the need for cautious and individually tailored approaches. Although some clinicians preferred gradual *vs* abrupt withdrawal of biologic treatments, others were concerned by the risk of immunogenicity to adalimumab and infliximab when tapering the dose or increasing the interval between administration. A study of etanercept in JIA found no difference between the two tapering approaches in terms of the flare risk [100].

Additionally, there is no general consensus regarding the decision of which medication to stop first, MTX or the biologic treatment, with some clinicians preferring to stop biologics for reasons related to cost and convenience, while others prioritize the withdrawal of the medication with the most side effects or associated with a poor safety profile as per a recent CARRA survey [101].

Several biomarkers, such as serum S100 proteins (e.g. myeloid-related protein MRP8/14 or calprotectin) and interleukin-18 (IL-18) [102, 103], along with imaging biomarkers, such as Power Doppler musculoskeletal ultrasound [104] and magnetic resonance imaging (MRI) [105], including whole body MRI [106, 107] have been proposed as promising candidates to help guide the DMARD tapering in JIA, while shorter duration from JIA onset and treatment initiation or before starting biologics for people who required biologics, longer sustained remission, absence of uveitis and certain JIA subtypes [99] were all associated with better chance of successful DMARD treatment cessation.

## Other treatments

Pilocarpine, a M1-M3 muscarinic receptor agonist, is recommended for use in cSjD associated with severe dryness by the BSR guideline across the life course [52] based on data available in adults, and dapaglifozin, a sodium-glucose cotransporter-2 inhibitor, can be used in children with LN and diabetes, based on KDIGO (Kidney Disease: Improving Global Outcomes) and EULAR recommendations for management of LN in adults [49, 50].

Sildenafil is a phosphodiesterase type 5 (PDE-5) inhibitor used off-license in CYP with severe Raynaud’s associated and pulmonary hypertension as per adult recommendation for use in systemic sclerosis [81].

Bosentan is a dual endothelin agonist licensed for use in children with idiopathic pulmonary arterial hypertension (PAH), used off-license in juvenile scleroderma associated with PAH, as well as severe Raynaud’s associated with digital ulcers [108].

Pamidronate and zolendronate, synthetic analogues of pyrophosphate, have been used off-license in CNO/CRMO with spinal lesions [24], as well as in the treatment of osteogenesis imperfecta [109] and juvenile osteoporosis [110, 111]. Zoledronate has been effective in decreasing the risk of fractures in children with GC-induced osteoporosis in a recent RCT [112].

Avacopan, a targeted synthetic complement 5a receptor (C5aR) antagonist, is recommended by the recently published BSR guideline for use in active AAV across the whole life-course to minimize the GC toxicity [16], although the paediatric dose is not established (trials ongoing).

The toxicity profile of DMARDs and the recommendations regarding their use during the conception, pregnancy and breastfeeding period, which may be relevant to older adolescents, have been reviewed recently [113].

## Transplant therapy in refractory paediatric RMDs

Allogeneic HSCT (allo-HSCT) is mainly reserved for the treatment of severe monogenic autoinflammatory diseases refractory to conventional treatments, with or without associated immune deficiency. Examples with good outcomes include deficiency of adenosine deaminase 2 associated with immunodeficiency [114] and mevalonate kinase deficiency [115], amongst others. Overall, there may be 10–20% mortality from the procedure, and outcomes are more favourable with good donor HLA match, limiting availability for some patients.

In JIA, allo-HSCT is mainly used for refractory sJIA [116], usually relapsing MAS (with or without refractory arthritis), or lung disease [117]. Results are encouraging, albeit with the aforementioned burden of morbidity associated with the procedure.

Autologous HSCT may still be considered for refractory non-systemic JIA [118] but is used less frequently in view of the emergence of newer drugs.

## Emerging treatments in paediatric rheumatology

There are currently many ongoing clinical trials in paediatric rheumatology, testing many of the therapies that have been developed or already proven successful in adult trials for corresponding rheumatological indications. We summarized in Table 4 all the treatments that are currently evaluated in paediatric clinical trials as available on the https://clinicaltrials.gov website as of 16 June 2025, including the indications for which they are tested, and the trial design and dose regimens explored. There is a plethora of bDMARDs and tsDMARDs currently explored in paediatric trials, in addition to new cell therapies, with the outcome of chimeric antigen receptor T-cell-based (CAR-T) therapies being probably the most eagerly awaited as they hold the promise of resetting the immune system and leading to disease cure. The majority of the clinical trials for bDMARDs and tsDMARDs are phase III clinical trials, with a minority of head-to-head paediatric clinical trials comparing TNFi with IL-17 and IL-23 blockade for treatment of ERA/JPsA.

**Table 4.** Ongoing clinical trials in paediatric populations with rheumatic conditions

Interventional medicinal product being tested	Mechanism of action	Trial registration reference	Phase	Indication	Paediatric population/dose regimens tested
cDMARDs *vs* standard of care					
Voclosporin VOCAL	Calcineurin inhibitor	NCT05288855 (completed)	Phase III	Lupus nephritis	12–18 years Different doses are tested: 15.8 mg BID, 23.7 mg BID and 31.6 mg BID, in addition to standard of care with mycophenolate mofetil and steroids
Voclosporin VOCAL-EXT	Calcineurin inhibitor	NCT05962788	Extension study Phase III	Lupus nephritis	As per dose decided by the VOCAL study
Small molecules *vs* standard of care					
Apremilast	PDA4 inhibition	NCT04804553 NCT05767047	Phase III Phase III	Active JPsA with inadequate response/intolerance≥ one DMARD (including bDMARDS) JPsA associated with oral ulcers	5 to <18 years No data available regarding the study dose/s
Avacopan	Complement 5a receptor (C5aR) antagonist	NCT06321601	Phase III	Active ANCA-associated vasculitis (AAV) Treatment used in combination with a rituximab or a cyclophosphamide-containing regimen.	6 years to <18 years of age
Bariticinib (JUVE-BASIS)	JAK 1/2 inhibition	NCT03773978 (completed)	Phase III	Active JIA (polyarticular, extended oligoarticular, enthesitis-related JIA, JPsA) with an inadequate response to at least one conventional or biologic DMARD.	2 years to <18 years 2 mg QD for children <9 years of age 4 mg QD for adolescent participants (12 to <18 years of age) and children ≥9 years of age
Baricitinib	JAK 1/2 inhibition	NCT03773965	Phase III	JIA patients including JPsA who have completed a previous baricitinib study (NCT03773978).	1 year to 18 years
Baricitinib	JAK 1 inhibition	NCT04088396	Phase III	sJIA with at least 2 active joints.	1 year to <18 years No data available regarding the study dose/s
Filgotinib	JAK 1 inhibition	NCT06222034	Phase I	JIA (polyarticular, extended oligoarticular, ERA, JPsA, sJIA with active arthritis without active systemic features or with stable active systemic features for at least 6 months).	8 years to 18 years old No data available regarding the study dose/s
Deucravacitinib	TYK2 inhibitor	NCT06869551	Phase III	Active JPsA with inadequate response/intolerance ≥ one csDMARD and/or bDMARD.	5 years to 17 years old No data available regarding the study dose/s
Upadacitinib (SELECT-YOUTH)	ATP‐competitive JAK inhibitor	NCT03725007	Phase I	Polyarticular course JIA (rheumatoid factor-positive or rheumatoid factor-negative polyarticular JIA, extended oligoarticular JIA, or systemic JIA with active arthritis and without active systemic features).	2 years to 17 years No data available regarding the study dose/s
Monoclonal antibodies *vs* standard of care					
Secukinumab	IL-17A blockade	NCT03769168	Phase III Extension study	JPsA/ERA patients included in the JUNIPERA trial. Long-term extension study.	Children and adults (≥2 years) Two dosing regimens studied, results under evaluation
Ixekizumab	IL-17A blockade	NCT04527380	Phase III	Active JPsA or ERA. Active comparator adalimumab.	2–17 years W > 50 kg to 80 mg SC Q4W, with a starting dose of 160 mg at Weeks 0, 4, 8 and 12; 25–50 kg to 40 mg SC Q4W; 10–25 kg to 20 mg SC Q4W.
Ustekinumab U-POPS	IL-12/23 blockade	NCT05252533	Phase I	JPsA, paediatric psoriasis prescribed at a dose indicated by their clinician. PK study.	Children (≥5 to <12 years) Adolescents (≥12 to < 18 years) Dosing regimen needs to be determined in paediatric population
Ustekinumab UNITED	IL-12/23 blockade	NCT05092269	Long-term extension study	JPsA patients who completed the dosing planned in the primary ustekinumab studies (CNTO1275CRD1001/PUC3001/CRD3004/JPA3001).	2 to <18 years of age No data available regarding the study dose/s
Ustekinumab OR Guselkumab PSUMMIT-Jr	IL-12/23 or IL-23 blockade	NCT05083182	Phase III	JPsA or ERA with inadequate response/intolerance≥1 DMARD (bDMARDs permitted, outside IL-23 inhibition).	5–17 years Dosing regimen needs to be determined in paediatric population
Certolizumab PASCAL	TNF blockade	NCT01550003	Phase III	Active polyarticular JIA (including JPsA) with inadequate response/intolerance≥1 DMARD (bDMARDs permitted).	2 to <18 years of age Two dosing regimen studied, results under evaluation
Sarilumab SKYPP	IL-6 blockade	NCT02776735	Open Label Phase II (trial completed)	Polyarticular-course JIA	2–17 years Dosing regimen needs to be determined in paediatric population
Anifrolumab	Type 1 IFN Receptor antagonist	NCT05835310	Phase III	Moderate to severe active SLE	5 to <18 years of age Dosing regimen needs to be determined in paediatric population.
Ianalumab SIRIUS-SLE 2	Dual mechanism of action: direct ADCC-mediated of B cells and BAFF-R blockade	NCT05624749	Phase III	Moderate to severe active SLE	12 to <18 years of age Dosing regimen needs to be determined in paediatric population
Head-to-head trials of monoclonal antibodies					
Risankizumab *vs* adalimumab	IL-23 *vs* TNF blockade	NCT06100744	Phase III	JPsA in patients who failed/were intolerant to ≥1 cDMARD.	5–18 years of age Risankizumab dosing regimen needs to be determined in paediatric population
Ixekizumab *vs* adalimumab	IL-17A *vs* TNF blockade	NCT04527380	Phase III	Active JPsA or ERA. Active comparator adalimumab.	2–17 years Ixekizumab dosing regimen needs to be determined in paediatric population
Cell-based therapies					
Chimeric antigen receptor (CAR) T-cell-based therapies	CD-19 CAR-T therapy	NCT06839976 NCT06904729	Single-centre, single-arm, open-label phase 1/2 studies open in various centres, such as Seattle, Philadelphia, USA; Great Ormond Street Hospital, London, UK; Guangzhou, China	Refractory cSLE; including both LN and non-renal cSLE.	12–18 years of age.
Chimeric antigen receptor(CAR) T-cell-based therapies	CD19 CAR-T therapy	NCT06569472	Phase I	Refractory JDM, intolerant or unresponsive to glucocorticoids and at least 2 immunosuppressants, rapidly progressive or associated with calcinosis and skin ulcers.	≥5 years and <17 years old.

Source: https://clinicaltrials.gov, accessed on 16 June 2025.

ADCC: antibody-dependent cellular cytotoxicity; BAFF-R: B-cell activating factor receptor; cSLE: childhood-onset systemic lupus erythematosus; ERA: enthesitis-related arthritis; IL: interleukin; JDM: juvenile dermatomyositis; JIA: juvenile idiopathic arthritis; JPsA: juvenile psoriatic arthritis; PK: pharmacokinetic; W: weight.

Ianalumab are the only monoclonal antibodies with a dual mechanism of action, targeting both BAFF and causing B-cell depletion through antibody-dependent cellular cytotoxicity currently tested in children with active cSLE, while T-cell engagers (CD19/CD3) are currently used in children with B-cell lineage malignancies in children [119] and are currently tested in adults with SLE.

The eagerly awaited clinical trials investigating the efficacy of CD19- CAR-T-cell-based therapies can offer children the opportunity to achieve long-term remission in various RMDs [8], although characterization of larger cohorts of individuals undergoing this type of treatment will provide the ultimate answer regarding their long-term efficacy and safety [120]. Compared with haematological malignancies, CD19 CAR-T seems to be better tolerated as no severe incidence of cytokine release syndrome (CRS) or immune-effector-cell-associated neurotoxicity syndrome (ICANS) have been reported [121]. Recently, a new type of self-limiting adverse reaction has been described, affecting 77% of individuals treated with CD19 CAR-T therapy, which will require further evaluation in larger cohorts [122].

The relatively high rate of relapse 12–24 month post CD19 CAR-T therapy in haematological malignancies [123], as well as the variable proportion of children achieving long-term remission off treatment with autologous or allogeneic transplant, associated with a more profound resetting of the immune system [124, 125] may suggest that achieving a permanent cure with CD19 CAR-T therapy in RMDs has relatively limited biological plausibility, although the preliminary reports of outcomes of young people with SLE are very encouraging [126]. Efforts to develop allogeneic CD19 CAR-T therapy may increase accessibility and decrease the costs in the future [127].

Several new therapies are currently tested in adult RMDs, and may be investigated in paediatric disease in the future: atacicept and talitacicept, TACI (transmembrane activator and calcium modulator and cyclophilin ligand interactor) recombinant fusion proteins that block both BAFF and APRIL (a proliferation-inducing ligand) are currently trialled in SLE, LN and IgA-related nephropathy in adults; ofatumumab (human mAb anti CD20) has been found efficacious in phase III trials in adult LN, and has been used off-label with success in cSLE [128]; brepocitinib (TYK2 and JAK1 inhibitor) was used off-label with success in refractory DM; ibrutinib, elsubrutinib, remibrutinib (Bruton tyrosine kinase-BTK inhibitors) and iscalimab/dazodalibep (anti-CD40mAb and anti-CD40L fusion protein) were associated with signals of efficacy in trials of adult SLE and SjD; nipocalimab/efgartigimod [anti-neonatal Fc receptor (FcRn) mAb that reduces circulating IgG] was associated with efficacy in preliminary studies in adult SLE/SjD.

The research advances in understanding the pathogenesis of monogenic lupus have open the possibility of new treatments, such as analogues of DNase1L3 (e.g. NTR-441, the first in class DNASE1L3 enzyme analogue), myeloid differentiation factor 88 (MyD88) inhibitors [129], as well as phosphoinositol 3 kinase (PI3K) and mTOR inhibitors [130], associated with therapeutic success in paediatric cases of LN [131]. Defects of endosomal nucleic acid sensing, associated with gain of function TRL7/9 mutations leading to cSLE-type phenotypes in children usually younger than 5 years, broadly defined as TLRopathies can be manipulated therapeutically by TLR7/8 inhibition [132]. Genetic abnormalities of the intracytoplasmic innate sensors, such as retinoic acid-inducible gene-I (RIG-I) and melanoma differentiation-associated protein 5 (MDA5) (RNA sensors), or cyclic GMP-AMP synthase (cGAS) that binds STING leading to the SAVI (sting associated vasculopathy with onset in infancy) phenotype associated with cSLE features (DNA sensor) can potentially be treated with JAKi [133] or STING/cGAS inhibitors [134].

Although not currently tested in trials in children, the IL-18 inhibition (e.g. with Tadekinig alfa, which is a recombinant IL-18 binding protein) has been investigated in severe autoinflammatory conditions characterized by IL-18 dysregulation. Tadekinig alfa is currently tested in adult Still’s disease [135] and has been used in compassionate settings for children with certain genetic mutations involving IL-18 signalling pathway, and if proven efficacious in clinical trials, could be added in the future to the therapeutic armamentarium for Still’s disease in children.

## Holistic and personalized care in paediatric rheumatology

Although this review has been focused on pharmacological interventions in paediatric rheumatology, we acknowledge the importance of non-pharmacological strategies, including physical and occupational therapy and exercise-based interventions, as well as psychological, behavioural, lifestyle and self-care strategies, underpinned by multidisciplinary, developmentally appropriate and holistic approaches to address the complex physical, psychological, educational, vocational and social aspects of RMDs, and support children and young people to live normal lives despite their conditions.

We recognize the emerging field of theragnostic, which aims to combine personalized diagnostic with the selection of the most appropriate therapies. The theragnostic armamentarium with potential clinical utility in paediatric rheumatology comprises many genetic tests, serum biomarkers (with S100 proteins and cytokine profiling being the most used), imaging biomarkers and validated outcomes measures describing well-defined disease states, in addition to therapeutic drug monitoring, pharmacogenomics and pharmacokinetic/pharmacodynamic modelling, which can all help facilitate the ‘molecular’ diagnosis of various paediatric RMD phenotypes, predict individuals more likely to respond to a certain therapy, monitor disease activity and treatment response, and if appropriate, help guide tapering strategies, as reviewed elsewhere [136].

## Conclusion

Clinicians and researchers in the field of paediatric rheumatology have witnessed the enormous progress that has been achieved in recent decades in expanding the pharmacological armamentarium used in the management of CYP with childhood-onset RMDs, which enabled unprecedented improvement in outcomes with broad societal benefit. Despite this, access to medications for children is still restricted in many parts of the world, which perpetuates health inequalities. Advances in genomics and precision medicine have facilitated early diagnosis and new therapeutic discoveries. However, the scarcity of approved therapeutic options, with evidence-based dosing regimens and established efficacy and safety in paediatric populations, highlights an ongoing unmet need. We advocate for accelerated approval of emerging paediatric therapies, facilitated by increased commitment to research, innovation and collaboration to match the progress seen in adult rheumatology. Pharmacological interventions should be integrated in a comprehensive care approach, promoting children’s overall health and wellbeing, ultimately aiming to preserve their quality of life and ensure their adequate access to the high-quality healthcare they deserve.

## Data Availability

No new data were generated or analysed in support of this article.

## References

[1] An J , Marwaha A, Laxer RM. Autoinflammatory diseases: a review. J Rheumatol 2024;51:848–61. 38879186 10.3899/jrheum.2023-1209

[2] Szekanecz Z , McInnes IB, Schett G et al Autoinflammation and autoimmunity across rheumatic and musculoskeletal diseases. Nat Rev Rheumatol 2021;17:585–95. 34341562 10.1038/s41584-021-00652-9

[3] Ozen S , Aksentijevich I. The past 25 years in paediatric rheumatology: insights from monogenic diseases. Nat Rev Rheumatol 2024;20:585–93. 39112602 10.1038/s41584-024-01145-1

[4] Ciurtin C , Jelusic M, Ozen S. Do we need distinct pediatric classification criteria for rheumatic diseases that affect both children and adults? Arthritis Rheumatol 2024;77:521–5. 10.1002/art.43058 PMC12039469 39542846

[5] Commissioning Medicines for Children in Specialised Services. https://www.england.nhs.uk/wp-content/uploads/2017/03/commissioning-medicines-for-children-in-specialised-services-v0.3.pdf (11 September 2025, date last accessed).

[6] Caplan AI. Mesenchymal stem cells: time to change the name!. Stem Cells Transl Med 2017;6:1445–51. 28452204 10.1002/sctm.17-0051 PMC5689741

[7] Jones OY , McCurdy D. Cell based treatment of autoimmune diseases in children. Front Pediatr 2022;10:855260. 35615628 10.3389/fped.2022.855260 PMC9124972

[8] Burki T. CAR T-cell therapy for SLE in children. Lancet Rheumatol 2024;6:e338. 10.1016/S2665-9913(24)00098-5 38642572

[9] Foster HE , Brogan PA (eds). Paediatric rheumatology, 2 edn. Oxford specialist handbooks in paediatrics. Oxford, UK: Oxford University Press, 2018. 10.1093/med/9780198738756.001.0001.

[10] Ohana O , Marmor I, Ferguson R et al Efficacy and safety of ibuprofen and naproxen in the treatment of oligoarticular juvenile idiopathic arthritis: bi-national cohort study. Immunopharmacol Immunotoxicol 2025;47:141–6. 39789705 10.1080/08923973.2024.2421523

[11] de Graeff N , Groot N, Ozen S et al European consensus-based recommendations for the diagnosis and treatment of Kawasaki disease—the SHARE initiative. Rheumatology (Oxford) 2019;58:672–82. 30535127 10.1093/rheumatology/key344

[12] Jone P-N , Tremoulet A, Choueiter N et al; American Heart Association Rheumatic Fever, Endocarditis, and Kawasaki Disease Committee of the Council on Lifelong Congenital Heart Disease and Heart Health in the Young; Council on Cardiovascular and Stroke Nursing; Council on Cardiovascular Radiology and Intervention; and Council on Clinical Cardiology. Update on Diagnosis and Management of Kawasaki Disease: a Scientific Statement From the American Heart Association. Circulation 2024;150:e481–e500. 39534969 10.1161/CIR.0000000000001295

[13] Groot N , de Graeff N, Marks SD et al European evidence-based recommendations for the diagnosis and treatment of childhood-onset lupus nephritis: the SHARE initiative. Ann Rheum Dis 2017;76:1965–73. 28877866 10.1136/annrheumdis-2017-211898

[14] Oldroyd AGS , Lilleker JB, Amin T et al; British Society for Rheumatology Standards, Audit and Guidelines Working Group. British Society for Rheumatology guideline on management of paediatric, adolescent and adult patients with idiopathic inflammatory myopathy. Rheumatology (Oxford) 2022;61:1760–8. 35355064 10.1093/rheumatology/keac115 PMC9398208

[15] de Graeff N , Groot N, Brogan P et al European consensus-based recommendations for the diagnosis and treatment of rare paediatric vasculitides—the SHARE initiative. Rheumatology (Oxford) 2020;59:919. 32073621 10.1093/rheumatology/keaa057

[16] Biddle K , Jade J, Wilson-Morkeh H et al; British Society for Rheumatology Guideline Steering Group. The 2025 British Society for Rheumatology management recommendations for ANCA-associated vasculitis. Rheumatology (Oxford) 2025;64:4463–9. 40499929 10.1093/rheumatology/keaf242 PMC12316360

[17] Fautrel B , Mitrovic S, De Matteis A et al EULAR/PReS recommendations for the diagnosis and management of Still’s disease, comprising systemic juvenile idiopathic arthritis and adult-onset Still’s disease. Ann Rheum Dis 2024;83:1614–27. 39317417 10.1136/ard-2024-225851 PMC11672000

[18] Shakoory B , Geerlinks A, Wilejto M et al; HLH/MAS Task Force. The 2022 EULAR/ACR points to consider at the early stages of diagnosis and management of suspected haemophagocytic lymphohistiocytosis/macrophage activation syndrome (HLH/MAS). Ann Rheum Dis 2023;82:1271–85. 37487610 10.1136/ard-2023-224123 PMC11017727

[19] Cleary G, McErlane F. https://gettingitrightfirsttime.co.uk/wp-content/uploads/2025/06/HLH-Guide-final-version-v1.3-June-2025.pdf (11 September 2025, date last accessed).

[20] Brogan P , Naden R, Ardoin SP et al The pediatric glucocorticoid toxicity index. Semin Arthritis Rheum 2022;56:152068. 35917759 10.1016/j.semarthrit.2022.152068

[21] Zhang E , Capponi S, Scobell R et al Real-world application of the pediatric Glucocorticoid Toxicity Index in childhood-onset lupus. Semin Arthritis Rheum 2024;68:152516. 39059156 10.1016/j.semarthrit.2024.152516 PMC11381140

[22] Chalhoub NE , Wenderfer SE, Levy DM et al; Childhood Arthritis and Rheumatology Research Alliance Lupus Nephritis Work Group and the Pediatric Rheumatology European Society Lupus Working Party. International consensus for the dosing of corticosteroids in childhood-onset systemic lupus erythematosus with proliferative lupus nephritis. Arthritis Rheumatol 2022;74:263–73. 34279063 10.1002/art.41930 PMC8766607

[23] Zulian F , Culpo R, Sperotto F et al Consensus-based recommendations for the management of juvenile localised scleroderma. Ann Rheum Dis 2019;78:1019–24. 30826775 10.1136/annrheumdis-2018-214697 PMC6691928

[24] Zhao Y , Wu EY, Oliver MS et al; Chronic Nonbacterial Osteomyelitis/Chronic Recurrent Multifocal Osteomyelitis Study Group and the Childhood Arthritis and Rheumatology Research Alliance Scleroderma, Vasculitis, Autoinflammatory and Rare Diseases Subcommittee. Consensus treatment plans for chronic nonbacterial osteomyelitis refractory to nonsteroidal antiinflammatory drugs and/or with active spinal lesions. Arthritis Care Res (Hoboken) 2018;70:1228–37. 29112802 10.1002/acr.23462 PMC5938153

[25] Ter Haar N , Lachmann H, Ozen S et al Treatment of autoinflammatory diseases: results from the Eurofever Registry and a literature review. Ann Rheum Dis 2013;72:678–85. 22753383 10.1136/annrheumdis-2011-201268

[26] Stahn C , Buttgereit F. Genomic and nongenomic effects of glucocorticoids. Nat Clin Pract Rheumatol 2008;4:525–33. 18762788 10.1038/ncprheum0898

[27] Rubin S , Ohana O, Goldberg O et al The efficacy and safety of intra-articular injection of triamcinolone acetonide versus triamcinolone hexacetonide for treatment of juvenile idiopathic arthritis. Pediatr Rheumatol Online J 2022;20:5. 35093116 10.1186/s12969-022-00666-x PMC8801083

[28] Foster HE , Brogan PA (eds) Paediatric rheumatology, 2 edn. Oxford Specialist Handbooks in Paediatrics. Oxford: Oxford Academic, 2018.

[29] Frid P , Augdal TA, Larheim TA et al Efficacy and safety of intraarticular corticosteroid injections in adolescents with juvenile idiopathic arthritis in the temporomandibular joint: a Norwegian 2-year prospective multicenter pilot study. Pediatr Rheumatol Online J 2020;18:75. 32998740 10.1186/s12969-020-00464-3 PMC7528594

[30] Resnick CM , Pedersen TK, Abramowicz S, Twilt M, Stoustrup PB. Time to reconsider management of the temporomandibular joint in juvenile idiopathic arthritis. J Oral Maxillofac Surg 2018;76:1145–6. 29567437 10.1016/j.joms.2018.02.019

[31] Stoustrup P , Resnick CM, Abramowicz S et al; Temporomandibular Joint Juvenile Arthritis Working Group. Management of Orofacial Manifestations of Juvenile Idiopathic Arthritis: interdisciplinary Consensus-Based Recommendations. Arthritis Rheumatol 2023;75:4–14. 36041065 10.1002/art.42338 PMC10100353

[32] Gotte AC. Intra-articular corticosteroids in the treatment of juvenile idiopathic arthritis: safety, efficacy, and features affecting outcome. A comprehensive review of the literature. Open Access Rheumatol 2009;1:37–49. 27789980 10.2147/oarrr.s5103 PMC5074724

[33] Onel KB , Horton DB, Lovell DJ et al 2021 American college of rheumatology guideline for the treatment of juvenile idiopathic arthritis: therapeutic approaches for oligoarthritis, temporomandibular joint arthritis, and systemic juvenile idiopathic arthritis. Arthritis Rheumatol 2022;74:553–69. 35233993 10.1002/art.42037 PMC10161784

[34] Ringold S , Angeles-Han ST, Beukelman T et al 2019 American college of rheumatology/arthritis foundation guideline for the treatment of juvenile idiopathic arthritis: therapeutic approaches for non-systemic polyarthritis, sacroiliitis, and enthesitis. Arthritis Rheumatol 2019;71:846–63. 31021537 10.1002/art.40884 PMC6561114

[35] Tillett W , Allen A, Tucker L et al Treatment of psoriatic arthritis with biologic and targeted synthetic DMARDs: british Society for Rheumatology guideline scope. Rheumatology (Oxford) 2021;60:1588–92. 33097948 10.1093/rheumatology/keaa526

[36] Lokhandwala S , Townsend J, Ciurtin C. Existing and Emerging Targeted Therapies in Juvenile Psoriatic Arthritis: challenges and Unmet Needs. Paediatr Drugs 2024;26:217–28. 38310623 10.1007/s40272-023-00618-2

[37] Constantin T , Foeldvari I, Anton J et al Consensus-based recommendations for the management of uveitis associated with juvenile idiopathic arthritis: the SHARE initiative. Ann Rheum Dis 2018;77:1107–17. 29592918 10.1136/annrheumdis-2018-213131 PMC6059050

[38] Giannini EH , Brewer EJ, Kuzmina N et al Methotrexate in resistant juvenile rheumatoid arthritis. Results of the U.S.A.-U.S.S.R. double-blind, placebo-controlled trial. The Pediatric Rheumatology Collaborative Study Group and The Cooperative Children’s Study Group. N Engl J Med 1992;326:1043–9. 1549149 10.1056/NEJM199204163261602

[39] Woo P , Southwood TR, Prieur A-M et al Randomized, placebo-controlled, crossover trial of low-dose oral methotrexate in children with extended oligoarticular or systemic arthritis. Arthritis Rheum 2000;43:1849–57. 10943876 10.1002/1529-0131(200008)43:8<1849::AID-ANR22>3.0.CO;2-F

[40] Foley CM , McKenna D, Gallagher K et al Systemic juvenile idiopathic arthritis: the Great Ormond Street Hospital experience (2005-2021). Front Pediatr 2023;11:1218312. 37780048 10.3389/fped.2023.1218312 PMC10536248

[41] Kearsley-Fleet L , Vicente González L, Steinke D et al; Biologics for Children with Rheumatic Diseases (BCRD) Study and the British Society for Paediatric and Adolescent Rheumatology Etanercept Cohort Study (BSPAR-ETN). Methotrexate persistence and adverse drug reactions in patients with juvenile idiopathic arthritis. Rheumatology (Oxford) 2019;58:1453–8. 30851113 10.1093/rheumatology/kez048 PMC6649753

[42] Hugle B , van Dijkhuizen EHP. MTX intolerance in children and adolescents with juvenile idiopathic arthritis. Rheumatology (Oxford) 2020;59:1482–8. 32259834 10.1093/rheumatology/keaa139

[43] Silverman E , Mouy R, Spiegel L et al; Leflunomide in Juvenile Rheumatoid Arthritis (JRA) Investigator Group. Leflunomide or methotrexate for juvenile rheumatoid arthritis. N Engl J Med 2005;352:1655–66. 15843668 10.1056/NEJMoa041810

[44] Silverman E , Spiegel L, Hawkins D et al Long-term open-label preliminary study of the safety and efficacy of leflunomide in patients with polyarticular-course juvenile rheumatoid arthritis. Arthritis Rheum 2005;52:554–62. 15693001 10.1002/art.20861

[45] van Rossum MAJ , van Soesbergen RM, Boers M et al; Dutch Juvenile Idiopathic Arthritis Study Group. Long-term outcome of juvenile idiopathic arthritis following a placebo-controlled trial: sustained benefits of early sulfasalazine treatment. Ann Rheum Dis 2007;66:1518–24. 17491099 10.1136/ard.2006.064717 PMC2111611

[46] Tynjälä P , Vähäsalo P, Tarkiainen M et al Aggressive combination drug therapy in very early polyarticular juvenile idiopathic arthritis (ACUTE-JIA): a multicentre randomised open-label clinical trial. Ann Rheum Dis 2011;70:1605–12. 21623000 10.1136/ard.2010.143347

[47] Kemper AR , Van Mater HA, Coeytaux RR, Williams Jr JW, Sanders GD. Systematic review of disease-modifying antirheumatic drugs for juvenile idiopathic arthritis. BMC Pediatr 2012;12:29. 22420649 10.1186/1471-2431-12-29 PMC3340294

[48] Yalamanchili P , Lee LY, Bushnell G et al Trends in new use of disease-modifying antirheumatic drugs for juvenile idiopathic arthritis among commercially insured children in the United States from 2001 to 2022. Arthritis Rheumatol 2025;77:468–76. 39435602 10.1002/art.43041 PMC11936494

[49] Kidney Disease: Improving Global Outcomes Lupus Nephritis Work Group. KDIGO 2024 Clinical Practice Guideline for the management of LUPUS NEPHRITIS. Kidney Int 2024;105:S1–S69. 38182286 10.1016/j.kint.2023.09.002

[50] Anders H-J , Loutan J, Bruchfeld A et al The management of lupus nephritis as proposed by EULAR/ERA 2019 versus KDIGO 2021. Nephrol Dial Transplant 2023;38:551–61. 34888694 10.1093/ndt/gfab351

[51] Brogan PA , Arch B, Hickey H et al Mycophenolate mofetil versus cyclophosphamide for remission induction in childhood polyarteritis nodosa: an open-label, randomized, Bayesian Noninferiority Trial. Arthritis Rheumatol 2021;73:1673–82. 33760371 10.1002/art.41730

[52] Price EJ , Benjamin S, Bombardieri M et al British Society for Rheumatology guideline on management of adult and juvenile onset Sjogren disease. Rheumatology (Oxford) 2025;64:409–39. 38621708 10.1093/rheumatology/keae152 PMC12013823

[53] Baughman RP , Valeyre D, Korsten P et al ERS clinical practice guidelines on treatment of sarcoidosis. Eur Respir J 2021;58:2004079. 34140301 10.1183/13993003.04079-2020

[54] Giles I , Allen A, Crossley A et al Prescribing anti-rheumatic drugs in pregnancy and breastfeeding-the British Society for Rheumatology guideline scope. Rheumatology (Oxford) 2021;60:3565–9. 33848327 10.1093/rheumatology/keab334

[55] Welzel T , Winskill C, Zhang N, Woerner A, Pfister M. Biologic disease modifying antirheumatic drugs and Janus kinase inhibitors in paediatric rheumatology—what we know and what we do not know from randomized controlled trials. Pediatr Rheumatol Online J 2021;19:46. 33766063 10.1186/s12969-021-00514-4 PMC7995584

[56] Brunner HI , Abud-Mendoza C, Viola DO et al Safety and efficacy of intravenous belimumab in children with systemic lupus erythematosus: results from a randomised, placebo-controlled trial. Ann Rheum Dis 2020;79:1340–8. 32699034 10.1136/annrheumdis-2020-217101 PMC7509523

[57] Brogan P , Yeung RSM, Cleary G et al; PePRS Study Group. Phase IIa Global Study Evaluating Rituximab for the Treatment of Pediatric Patients With Granulomatosis With Polyangiitis or Microscopic Polyangiitis. Arthritis Rheumatol 2022;74:124–33. 34164952 10.1002/art.41901 PMC9299798

[58] Acharya NR , Ramanan AV, Coyne AB et al; ADJUST Study Group. Stopping of adalimumab in juvenile idiopathic arthritis-associated uveitis (ADJUST): a multicentre, double-masked, randomised controlled trial. Lancet 2025;405:303–13. 39863370 10.1016/S0140-6736(24)02468-1 PMC13398007

[59] Kearsley-Fleet L , Baildam E, Beresford MW et al Successful stopping of biologic therapy for remission in children and young people with juvenile idiopathic arthritis. Rheumatology (Oxford) 2023;62:1926–35. 36104094 10.1093/rheumatology/keac463 PMC10152290

[60] Choida V , Hall-Craggs M, Jebson BR et al Biomarkers of Response to Biologic Therapy in Juvenile Idiopathic Arthritis. Front Pharmacol 2020;11:635823. 33603671 10.3389/fphar.2020.635823 PMC7884612

[61] Wedderburn LR , Ramanan AV, Croft AP et al; CLUSTER Consortium. Towards molecular-pathology informed clinical trials in childhood arthritis to achieve precision medicine in juvenile idiopathic arthritis. Ann Rheum Dis 2023;82:449–56. 36600186 10.1136/ard-2022-222553 PMC10086280

[62] Parikh CR , Ponnampalam JK, Seligmann G et al Impact of immunogenicity on clinical efficacy and toxicity profile of biologic agents used for treatment of inflammatory arthritis in children compared to adults. Ther Adv Musculoskelet Dis 2021;13:1759720X211002685. 10.1177/1759720X211002685 PMC8212384 34188697

[63] Kawano-Dourado L , Kristianslund EK, Zeraatkar D et al Proactive therapeutic drug monitoring of biologic drugs in adult patients with inflammatory bowel disease, inflammatory arthritis, or psoriasis: a clinical practice guideline. BMJ 2024;387:e079830. 39467592 10.1136/bmj-2024-079830

[64] Yeo L , Naheed A, Richards C, Ciurtin C. Childhood-onset ANCA-associated vasculitis: from genetic studies to advances in pathogenesis, classification and novel therapeutic approaches. Int J Mol Sci 2024;25:13704. 39769465 10.3390/ijms252413704 PMC11676361

[65] Davies R , Gaynor D, Hyrich KL, Pain CE. Efficacy of biologic therapy across individual juvenile idiopathic arthritis subtypes: a systematic review. Semin Arthritis Rheum 2017;46:584–93. 27914689 10.1016/j.semarthrit.2016.10.008

[66] Lovell DJ , Ruperto N, Goodman S et al; Pediatric Rheumatology International Trials Organisation. Adalimumab with or without methotrexate in juvenile rheumatoid arthritis. N Engl J Med 2008;359:810–20. 18716298 10.1056/NEJMoa0706290

[67] Brunner HI , Ruperto N, Tzaribachev N et al; Paediatric Rheumatology International Trials Organisation (PRINTO) and the Pediatric Rheumatology Collaborative Study Group (PRCSG). Subcutaneous golimumab for children with active polyarticular-course juvenile idiopathic arthritis: results of a multicentre, double-blind, randomised-withdrawal trial. Ann Rheum Dis 2018;77:21–9. 28507219 10.1136/annrheumdis-2016-210456 PMC5754736

[68] Lovell DJ , Giannini EH, Reiff A et al Etanercept in children with polyarticular juvenile rheumatoid arthritis. Pediatric Rheumatology Collaborative Study Group. N Engl J Med 2000;342:763–9. 10717011 10.1056/NEJM200003163421103

[69] Liu DW , Chen JJ, Tang XM, Zhang Y, Zhou J. Infliximab therapy and outcomes in patients with polyarticular juvenile idiopathic arthritis: a single-center study in China. World J Pediatr 2020;16:68–73. 31612428 10.1007/s12519-019-00316-5

[70] Ozen S , Sağ E, Oton T et al EULAR/PReS endorsed recommendations for the management of familial Mediterranean fever (FMF): 2024 update. Ann Rheum Dis 2025;84:899–909. 40234174 10.1016/j.ard.2025.01.028

[71] De Benedetti F , Gattorno M, Anton J et al Canakinumab for the treatment of autoinflammatory recurrent fever syndromes. N Engl J Med 2018;378:1908–19. 29768139 10.1056/NEJMoa1706314

[72] Hansmann S , Lainka E, Horneff G et al Consensus protocols for the diagnosis and management of the hereditary autoinflammatory syndromes CAPS, TRAPS and MKD/HIDS: a German PRO-KIND initiative. Pediatr Rheumatol Online J 2020;18:17. 32066461 10.1186/s12969-020-0409-3 PMC7027082

[73] Brunner HI , Ruperto N, Ramanan AV et al; PRINTO and PRCSG Investigators. Long-term efficacy and safety of subcutaneous tocilizumab in clinical trials of polyarticular or systemic juvenile idiopathic arthritis. Rheumatology (Oxford 2024;63:2535–46. ). 38552315 10.1093/rheumatology/keae180 PMC11371380

[74] Brunner HI , Foeldvari I, Alexeeva E et al; Paediatric Rheumatology INternational Trials Organization (PRINTO) and Pediatric Rheumatology Collaborative Study Group (PRCSG). Secukinumab in enthesitis-related arthritis and juvenile psoriatic arthritis: a randomised, double-blind, placebo-controlled, treatment withdrawal, phase 3 trial. Ann Rheum Dis 2023;82:154–60. 35961761 10.1136/ard-2022-222849 PMC9811076

[75] Leu JH , Shiff NJ, Clark M et al Intravenous golimumab in patients with polyarticular juvenile idiopathic arthritis and juvenile psoriatic arthritis and subcutaneous ustekinumab in patients with juvenile psoriatic arthritis: extrapolation of data from studies in adults and adjacent pediatric populations. Paediatr Drugs 2022;24:699–714. 36171515 10.1007/s40272-022-00533-y PMC9592662

[76] Lovell DJ , Tzaribachev N, Henrickson M et al; for PRINTO and the Pediatric Rheumatology Collaborative Study Group (PRCSG)§. Safety and effectiveness of abatacept in juvenile idiopathic arthritis: results from the PRINTO/PRCSG registry. Rheumatology (Oxford) 2024;63:SI195–SI206. 38243722 10.1093/rheumatology/keae025 PMC11381685

[77] Kearsley-Fleet L , Sampath S, McCann LJ et al Use and effectiveness of rituximab in children and young people with juvenile idiopathic arthritis in a cohort study in the United Kingdom. Rheumatology (Oxford) 2019;58:331–5. 30358861 10.1093/rheumatology/key306 PMC6343463

[78] Kearsley-Fleet L , Baildam E, Beresford MW et al; UK JIA Biologics Register. Outcomes after anti-tumour necrosis factor originator to biosimilar switching in children and young people with juvenile idiopathic arthritis in the UK: a national cohort study. Lancet Rheumatol 2024;6:e438–e46. 38843858 10.1016/S2665-9913(24)00087-0

[79] Biddle K , Jade J, Wilson-Morkeh H et al; British Society for Rheumatology Guideline Steering Group. Executive summary: the 2025 British Society for Rheumatology management recommendations for ANCA-associated vasculitis. Rheumatology (Oxford) 2025;64:4463–9. 40499929 10.1093/rheumatology/keaf242 PMC12316360

[80] Fanouriakis A , Kostopoulou M, Andersen J et al EULAR recommendations for the management of systemic lupus erythematosus: 2023 update. Ann Rheum Dis 2024;83:15–29. 37827694 10.1136/ard-2023-224762

[81] Del Galdo F , Lescoat A, Conaghan PG et al EULAR recommendations for the treatment of systemic sclerosis: 2023 update. Ann Rheum Dis 2025;84:29–40. 39874231 10.1136/ard-2024-226430

[82] De Benedetti F , Grom AA, Brogan PA et al Efficacy and safety of emapalumab in macrophage activation syndrome. Ann Rheum Dis 2023;82:857–65. 37001971 10.1136/ard-2022-223739 PMC10314091

[83] Cetin Gedik K , Lamot L, Romano M et al The 2021 European Alliance of Associations for Rheumatology/American College of Rheumatology points to consider for diagnosis and management of autoinflammatory type I interferonopathies: CANDLE/PRAAS, SAVI, and AGS. Arthritis Rheumatol 2022;74:735–51. 35315249 10.1002/art.42087

[84] Kretzschmar G , Páez LP, Tan Z et al Normalized interferon signatures and clinical improvements by IFNAR1 blocking antibody (anifrolumab) in patients with type I interferonopathies. J Clin Immunol 2024;45:31. 39441221 10.1007/s10875-024-01826-2 PMC11499543

[85] Davies HD ; Committee On Infectious D. Infectious complications with the use of biologic response modifiers in infants and children. Pediatrics 2016;138:e20161209. 10.1542/peds.2016-1209 27432853

[86] Jansen MHA , Rondaan C, Legger GE et al EULAR/PRES recommendations for vaccination of paediatric patients with autoimmune inflammatory rheumatic diseases: update 2021. Ann Rheum Dis 2023;82:35–47. 35725297 10.1136/annrheumdis-2022-222574

[87] Davis JS , Ferreira D, Paige E, Gedye C, Boyle M. Infectious complications of biological and small molecule targeted immunomodulatory therapies. Clin Microbiol Rev 2020;33:e00035–19. 32522746 10.1128/CMR.00035-19 PMC7289788

[88] Henderson C , Goldbach-Mansky R. Monogenic IL-1 mediated autoinflammatory and immunodeficiency syndromes: finding the right balance in response to danger signals. Clin Immunol 2010;135:210–22. 20353899 10.1016/j.clim.2010.02.013 PMC2856775

[89] Poli MC , Aksentijevich I, Bousfiha AA et al Human inborn errors of immunity: 2024 update on the classification from the International Union of Immunological Societies Expert Committee. J Hum Immun 2025;1:e20250003. 10.70962/jhi.20250003 PMC12829761 41608114

[90] Ruperto N , Brunner HI, Synoverska O et al; Paediatric Rheumatology International Trials Organisation (PRINTO) and Pediatric Rheumatology Collaborative Study Group (PRCSG). Tofacitinib in juvenile idiopathic arthritis: a double-blind, placebo-controlled, withdrawal phase 3 randomised trial. Lancet 2021;398:1984–96. 34767764 10.1016/S0140-6736(21)01255-1

[91] Ramanan AV , Quartier P, Okamoto N et al; Paediatric Rheumatology International Trials Organisation. Baricitinib in juvenile idiopathic arthritis: an international, phase 3, randomised, double-blind, placebo-controlled, withdrawal, efficacy, and safety trial. Lancet 2023;402:555–70. 37423231 10.1016/S0140-6736(23)00921-2

[92] Brunner HI , Shmagel A, Horneff G et al Pharmacokinetics, efficacy, and safety of upadacitinib in pediatric patients with polyarticular-course juvenile idiopathic arthritis: an interim analysis of an open-label, phase 1 trial. Arthritis Care Res (Hoboken) 2025;77:584–93. 39542836 10.1002/acr.25465 PMC12038220

[93] Ramanan AV , Guly CM, Keller SY et al Clinical effectiveness and safety of baricitinib for the treatment of juvenile idiopathic arthritis-associated uveitis or chronic anterior antinuclear antibody-positive uveitis: study protocol for an open-label, adalimumab active-controlled phase 3 clinical trial (JUVE-BRIGHT). Trials 2021;22:689. 34627340 10.1186/s13063-021-05651-5 PMC8502273

[94] Boyadzhieva Z , Ruffer N, Burmester G, Pankow A, Krusche M. Effectiveness and safety of JAK inhibitors in autoinflammatory diseases: a systematic review. Front Med (Lausanne) 2022;9:930071. 35833101 10.3389/fmed.2022.930071 PMC9271622

[95] Sanchez GAM , Reinhardt A, Ramsey S et al JAK1/2 inhibition with baricitinib in the treatment of autoinflammatory interferonopathies. J Clin Invest 2018;128:3041–52. 29649002 10.1172/JCI98814 PMC6026004

[96] Ll Wilkinson MG , Deakin CT, Papadopoulou C, Eleftheriou D, Wedderburn LR. JAK inhibitors: a potential treatment for JDM in the context of the role of interferon-driven pathology. Pediatr Rheumatol Online J 2021;19:146. 34563217 10.1186/s12969-021-00637-8 PMC8466894

[97] Papadopoulou C , Hong Y, Omoyinmi E, Brogan PA, Eleftheriou D. Janus kinase 1/2 inhibition with baricitinib in the treatment of juvenile dermatomyositis. Brain 2019;142:e8. 30715143 10.1093/brain/awz005 PMC6391598

[98] Papadopoulou C , Martin N, Rafiq N et al Elicitation of expert prior opinion to design the BARJDM trial in juvenile dermatomyositis. Rheumatology (Oxford) 2024;63:3271–8. 39073903 10.1093/rheumatology/keae392 PMC11637550

[99] Halyabar O , Mehta J, Ringold S, Rumsey DG, Horton DB. Treatment withdrawal following remission in juvenile idiopathic arthritis: a systematic review of the literature. Paediatr Drugs 2019;21:469–92. 31673960 10.1007/s40272-019-00362-6 PMC7301222

[100] Pratsidou-Gertsi P , Trachana M, Pardalos G, Kanakoudi-Tsakalidou F. A follow-up study of patients with juvenile idiopathic arthritis who discontinued etanercept due to disease remission. Clin Exp Rheumatol 2010;28:919–22. 21205467

[101] Currie GR , Pham T, Twilt M et al Perspectives of pediatric rheumatologists on initiating and tapering biologics in patients with juvenile idiopathic arthritis: a formative qualitative study. Patient 2022;15:599–609. 35322390 10.1007/s40271-022-00575-x

[102] Holzinger D , Frosch M, Kastrup A et al The Toll-like receptor 4 agonist MRP8/14 protein complex is a sensitive indicator for disease activity and predicts relapses in systemic-onset juvenile idiopathic arthritis. Ann Rheum Dis 2012;71:974–80. 22267331 10.1136/annrheumdis-2011-200598

[103] Shimizu M , Nakagishi Y, Yoshida A, Yachie A. Serum interleukin 18 as a diagnostic remission criterion in systemic juvenile idiopathic arthritis. J Rheumatol 2014;41:2328–30. 25362722 10.3899/jrheum.140025

[104] Miotto ESVB , Mitraud SAV, Furtado RNV et al Patients with juvenile idiopathic arthritis in clinical remission with positive power Doppler signal in joint ultrasonography have an increased rate of clinical flare: a prospective study. Pediatr Rheumatol Online J 2017;15:80. 29132381 10.1186/s12969-017-0208-7 PMC5683235

[105] Mazzoni M , Pistorio A, Magnaguagno F et al Predictive value of magnetic resonance imaging in patients with juvenile idiopathic arthritis in clinical remission. Arthritis Care Res (Hoboken) 2023;75:198–205. 34286915 10.1002/acr.24757 PMC10087925

[106] Choida V , Bray TJP, van Vucht N et al Detection of inflammation by whole-body MRI in young people with juvenile idiopathic arthritis. Rheumatology (Oxford) 2024;63:SI207–SI214. 38244609 10.1093/rheumatology/keae039 PMC11381681

[107] Ciurtin C , Bray T, Choida V, Hall-Craggs MA. Whole-body MRI for juvenile idiopathic arthritis. Lancet Rheumatol 2023;5:e6–e8. 38251509 10.1016/S2665-9913(22)00342-3

[108] Roldan R , Morote G, Castro Mdel C et al Efficacy of bosentan in treatment of unresponsive cutaneous ulceration in disabling pansclerotic morphea in children. J Rheumatol 2006;33:2538–40. 17143989

[109] Marom R , Rabenhorst BM, Morello R. Osteogenesis imperfecta: an update on clinical features and therapies. Eur J Endocrinol 2020;183:R95–R106. 32621590 10.1530/EJE-20-0299 PMC7694877

[110] Ward LM , Weber DR, Munns CF, Högler W, Zemel BS. A contemporary view of the definition and diagnosis of osteoporosis in children and adolescents. J Clin Endocrinol Metab 2020;105:e2088–97. 31865390 10.1210/clinem/dgz294 PMC7121121

[111] Ward LM. A practical guide to the diagnosis and management of osteoporosis in childhood and adolescence. Front Endocrinol (Lausanne) 2023;14:1266986. 38374961 10.3389/fendo.2023.1266986 PMC10875302

[112] Ward LM , Choudhury A, Alos N et al Zoledronic acid vs placebo in pediatric glucocorticoid-induced osteoporosis: a randomized, double-blind, phase 3 trial. J Clin Endocrinol Metab 2021;106:e5222–35. 34228102 10.1210/clinem/dgab458

[113] Russell MD , Dey M, Flint J et al; BSR Standards, Audit and Guidelines Working Group. British Society for Rheumatology guideline on prescribing drugs in pregnancy and breastfeeding: immunomodulatory anti-rheumatic drugs and corticosteroids. Rheumatology (Oxford) 2023;62:e48–e88. 36318966 10.1093/rheumatology/keac551 PMC10070073

[114] Lee PY , Davidson BA, Abraham RS et al; DADA2 Foundation. Evaluation and management of deficiency of adenosine deaminase 2: an international consensus statement. JAMA Netw Open 2023;6:e2315894. 37256629 10.1001/jamanetworkopen.2023.15894

[115] Lengvári L , Takács K, Lengyel A et al Mevalonate kinase deficiency: an updated clinical overview and revision of the SHARE recommendations. Front Immunol 2024;15:1466844. 39600705 10.3389/fimmu.2024.1466844 PMC11590122

[116] M F Silva J , Ladomenou F, Carpenter B et al Allogeneic hematopoietic stem cell transplantation for severe, refractory juvenile idiopathic arthritis. Blood Adv 2018;2:777–86. 29618462 10.1182/bloodadvances.2017014449 PMC5894259

[117] Matt MG , Drozdov D, Bendstrup E et al Allogeneic haematopoietic stem-cell transplantation for children with refractory systemic juvenile idiopathic arthritis and associated lung disease: outcomes from an international, retrospective cohort study. Lancet Rheumatol 2025;7:e243–e51. 39718183 10.1016/S2665-9913(24)00275-3 PMC11949720

[118] De Kleer IM , Brinkman DMC, Ferster A et al Autologous stem cell transplantation for refractory juvenile idiopathic arthritis: analysis of clinical effects, mortality, and transplant related morbidity. Ann Rheum Dis 2004;63:1318–26. 15361393 10.1136/ard.2003.017798 PMC1754760

[119] Lyons KU , Gore L. Bispecific T-cell engagers in childhood B-acute lymphoblastic leukemia. Haematologica 2024;109:1668–76. 38832422 10.3324/haematol.2023.283818 PMC11141658

[120] Mullard A. CAR T cell therapies raise hopes—and questions—for lupus and autoimmune disease. Nat Rev Drug Discov 2023;22:859–61. 37833555 10.1038/d41573-023-00166-x

[121] Müller F , Taubmann J, Bucci L et al CD19 CAR T-cell therapy in autoimmune disease—a case series with follow-up. N Engl J Med 2024;390:687–700. 38381673 10.1056/NEJMoa2308917

[122] Hagen M , Müller F, Wirsching A et al Local immune effector cell-associated toxicity syndrome in CAR T-cell treated patients with autoimmune disease: an observational study. Lancet Rheumatol 2025;7:e424–33s. 40318690 10.1016/S2665-9913(25)00091-8

[123] Cappell KM , Kochenderfer JN. Long-term outcomes following CAR T cell therapy: what we know so far. Nat Rev Clin Oncol 2023;20:359–71. 37055515 10.1038/s41571-023-00754-1 PMC10100620

[124] Abinun M , Slatter MA. Haematopoietic stem cell transplantation in paediatric rheumatic disease. Curr Opin Rheumatol 2021;33:387–97. 34261117 10.1097/BOR.0000000000000823

[125] Satirer O , Henes JC, Doring M et al Autologous haematopoiesis stem cell transplantation (AHSCT) for treatment-refractory autoimmune diseases in children. RMD Open 2024;10:e004381. 10.1136/rmdopen-2024-004381 PMC11253738 39004431

[126] Mackensen A , Müller F, Mougiakakos D et al Anti-CD19 CAR T cell therapy for refractory systemic lupus erythematosus. Nat Med 2022;28:2124–32. 36109639 10.1038/s41591-022-02017-5

[127] Lonez C , Breman E. Allogeneic CAR-T therapy technologies: has the promise been met? Cells 2024;13:146. 38247837 10.3390/cells13020146 PMC10814647

[128] Cinar OK , Marlais M, Al Obaidi M et al Ofatumumab use in juvenile systemic lupus erythematosus: A single centre experience. Lupus 2021;30:527–30. 33327846 10.1177/0961203320981137

[129] Tilstra JS , Kim M, Gordon RA et al B cell-intrinsic Myd88 regulates disease progression in murine lupus. J Exp Med 2023;220:e20230263. 10.1084/jem.20230263 PMC10541815 37787782

[130] Fasano S , Milone A, Nicoletti GF, Isenberg DA, Ciccia F. Precision medicine in systemic lupus erythematosus. Nature Reviews Rheumatology 2023;19:331–42. 37041269 10.1038/s41584-023-00948-y

[131] Marchi-Silva R , Martins DAB, Carolina LA et al New Insights on childhood lupus nephritis. Int J Nephrol Renovasc Dis 2025;18:1–12. 39829960 10.2147/IJNRD.S405789 PMC11740589

[132] Felten R , Scherlinger M, Mertz P, Chasset F, Arnaud L. New biologics and targeted therapies in systemic lupus: from new molecular targets to new indications. A systematic review. Joint Bone Spine 2023;90:105523. 36623799 10.1016/j.jbspin.2023.105523

[133] Wu J , Zhou Q, Zhou H, Lu M. Case report: JAK1/2 inhibition with baricitinib in the treatment of STING-associated vasculopathy with onset in infancy. Pediatr Rheumatol Online J 2023;21:131. 37884945 10.1186/s12969-023-00916-6 PMC10601276

[134] Decout A , Katz JD, Venkatraman S, Ablasser A. The cGAS–STING pathway as a therapeutic target in inflammatory diseases. Nat Rev Immunol 2021;21:548–69. 33833439 10.1038/s41577-021-00524-z PMC8029610

[135] Gabay C , Fautrel B, Rech J et al Open-label, multicentre, dose-escalating phase II clinical trial on the safety and efficacy of tadekinig alfa (IL-18BP) in adult-onset Still’s disease. Ann Rheum Dis 2018;77:840–7. 29472362 10.1136/annrheumdis-2017-212608 PMC5965361

[136] Balevic SJ , Sagcal-Gironella ACP. Precision medicine: towards individualized dosing in pediatric rheumatology. Rheum Dis Clin North Am 2022;48:305–30. 34798954 10.1016/j.rdc.2021.09.010

