# Correction to: The evolving landscape of rheumatic drugs and their indications in paediatric and adolescent care

**DOI:** 10.1093/rheumatology/keag245

**Published:** 2026-05-31

This is a correction to: Coziana Ciurtin, Mihaela Sparchez, Despina Eleftheriou, Paul Brogan, The evolving landscape of rheumatic drugs and their indications in paediatric and adolescent care, *Rheumatology*, Volume 64, Issue 12, December 2025, Pages 6048–6070, https://doi.org/10.1093/rheumatology/keaf455

In the originally published version of this manuscript, author name Despina Eleftheriou was misspelled. This error has been corrected.

